# Histone Acetyltransferase CfGcn5-Mediated Autophagy Governs the Pathogenicity of *Colletotrichum fructicola*

**DOI:** 10.1128/mbio.01956-22

**Published:** 2022-08-17

**Authors:** Shengpei Zhang, Yuan Guo, Sizheng Li, He Li

**Affiliations:** a College of Forestry, Central South University of Forestry and Technology, Changsha, China; b Key Laboratory of National Forestry, Grassland Administration on Control of Artificial Forest Diseases and Pests in South China, Changsha, China; c Hunan Provincial Key Laboratory for Control of Forest Diseases and Pests, Changsha, China; d Key Laboratory for Non-wood Forest Cultivation and Conservation of Ministry of Education, Changsha, China; Universidad de Córdoba

**Keywords:** histone acetyltransferase, autophagy, degradation, pathogenicity, *C. fructicola*

## Abstract

*Camellia oleifera* is a woody edible-oil plant in China, and anthracnose occurs wherever it is grown, causing serious losses each year. We previously identified that the histone acetyltransferase CfGcn5 orchestrates growth, development, and pathogenicity in *Colletotrichum fructicola*, the major causal agent of anthracnose on *C. oleifera*. To elucidate the underlying mechanism, we conducted a transcriptome analysis and found that CfGcn5 is mainly involved in ribosomes, catalytic and metabolic processes, primary metabolism, and autophagy. In addition, we provided evidence showing that CfGcn5 serves as an autophagy repressor to mediate the expression of many autophagy-related genes (*ATG*) and undergoes degradation during autophagy. Moreover, we found that the *CfATG8* and *CfATG9* gene-deletion mutants had defects in mitosis and autophagy, resulting in their decreased appressoria formation rates and lower turgor pressure. These combined effects caused the failure of their appressoria functions and caused defects on their pathogenicity, revealing the importance of autophagy in pathogenicity. Taken together, our study illustrates that the autophagy repressor CfGcn5 undergoes degradation in order to regulate autophagy-dependent pathogenicity in *C. fructicola*.

## INTRODUCTION

*Camellia oleifera* is a woody, edible-oil plant native to China, and it has been widely grown in southern China for more than 2,000 years (1). Owing to the high content of monounsaturated fatty acid in the edible oil extracted from *C. oleifera* seeds, the oil is beneficial to human health and is popular in Chinese cooking (2, 3). It is also utilized by the cosmetic industry in the U.S.A., Japan, and France (4). Although the plantations for *C. oleifera* reached 4.39 million hectares and the oil yield was up to 750 kg/ha, it is still not enough to meet consumers’ demands (1, 5). One of the major limiting factors is a devastating disease called anthracnose, which commonly occurs on *C. oleifera* (6). We previously revealed that *Colletotrichum fructicola* is the major causal agent of anthracnose on *C. oleifera* (6). We also demonstrated that the histone acetyltransferase CfGcn5 orchestrates growth, conidiation, and pathogenicity in *C. fructicola* and that the nucleus localization of CfGcn5 is essential, but not sufficient, for its full function (7). How CfGcn5 functions in the nucleus remains unknown.

Gcn5 (general control nonderepressible 5) was originally identified from yeast mutants that showed defects in amino acid synthesis (8, 9), and it was proven to be a regulator for gene transcription (8). Then, its homolog p55 in *Tetrahymena* was demonstrated to be a nuclear histone acetyltransferase (HAT), which established the link between histone acetylation and gene activation (10, 11). Subsequently, Gcn5 was shown to target specific lysine residues in histones H3 and H2B to epigenetically regulate global gene transcription (12–16). There is limited evidence supporting the importance of Gcn5-regulated gene transcription in the development and pathogenicity of filamentous fungi. In Aspergillus nidulans, the Gcn5 homolog AnGcnE regulates the expression of conidiation-related genes and secondary metabolism, thereby mediating conidiation (17). In Fusarium graminearum, FgGcn5 regulates the expression of many transcription factors and virulence-associated genes, thereby mediating pathogenicity (18). Despite these findings, the genome-wide gene expression regulatory mechanism of such a nucleus-localized protein in forestry phytopathogens, including *C. fructicola*, remains unknown.

In the past 2 decades, accumulated evidences has supported the pivotal regulatory function of acetylation for autophagy through transcriptional and posttranscriptional regulation (19, 20). For example, the upregulation of *ATG7* is accompanied by the hyperacetylation of its promoter region when autophagy happens in yeast cells (21). The acetylation of Forkhead box O (FoxO) transcription factors, which are the transcription factors of autophagy-related genes (*ATG*), controls autophagy by regulating the *ATG* transcripts in mammalian cells (22, 23). Also, multiple Atg proteins are known to change their acetylation levels during autophagy (24–26).

Autophagy is a conserved and pivotal process in which proteins and organelles are degraded and recycled in vacuoles (lysosomes) according to energetic and functional demands under cellular differentiation, tissue remodeling, and environmental stress (27, 28). Multiple lines of evidence have emerged that autophagy is a crucial process for virulence in plant-pathogenic fungi (29–31), and they have further established the link between acetylation and autophagy-dependent pathogenicity in Magnaporthe oryzae and F. graminearum (32–36).

Although the functions of autophagy in pathogenicity have been apparent in M. oryzae and in F. graminearum, little is known about autophagy and the relationship between acetylation and autophagy in *Colletotrichum* spp., which is ranked in the top 10 plant fungal pathogens (37). Here, we identified that CfGcn5 regulates global gene expression, including many *ATG* genes, and that CfGcn5 is an autophagy repressor that undergoes degradation during autophagy in *C. fructicola*. In addition, we demonstrated that CfAtg8 and CfAtg9 are required for pathogenicity due to their participation in autophagy and mitosis. Taken together, we revealed that CfGcn5 is an autophagy repressor that undergoes degradation in order to regulate autophagy-dependent pathogenicity in *C. fructicola*.

## RESULTS

### Transcriptome analysis of the genes regulated by CfGcn5.

**FIG 1:**
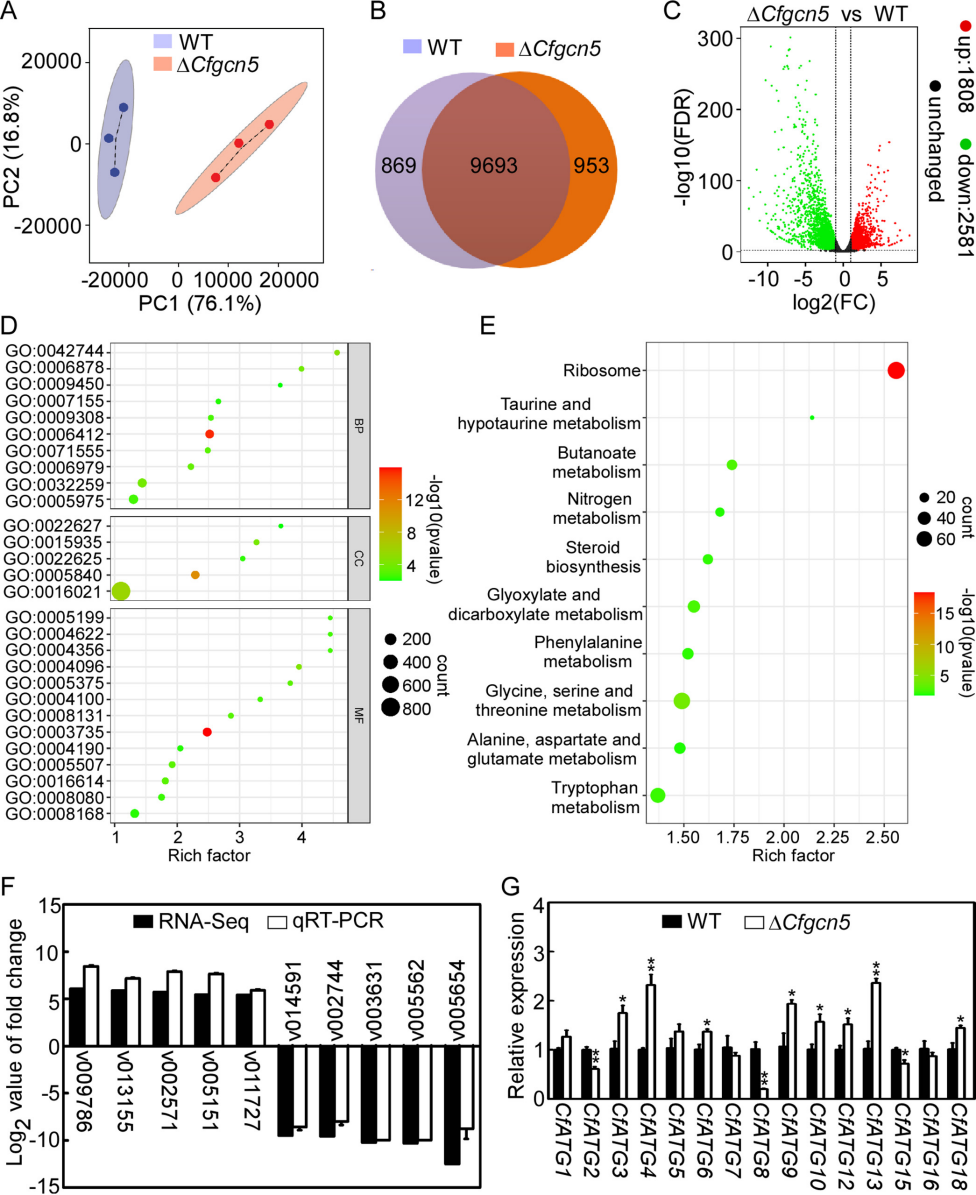
The gene expression data between the WT and Δ*Cfgcn5* mutant. (A) Principal-component analysis of the WT and Δ*Cfgcn5* mutant. (B) Global view of the gene expression levels in the WT and Δ*Cfgcn5* mutant. (C) Volcano plot of DEGs between the WT and Δ*Cfgcn5* mutant. Red dots represent upregulated genes, and green dots represent downregulated genes. (D) Gene ontology enrichment of the DEGs (*P* < 0.01). The BP (biological process) includes GO:0042744 (hydrogen peroxide catabolic process), GO:0006878 (cellular copper ion homeostasis), GO:0009450 (gamma-aminobutyric acid catabolic process), GO:0007155 (cell adhesion), GO:0009308 (amine metabolic process), GO:0006412(translation), GO:0071555 (cell wall organization), GO:0006979 (response to oxidative stress), GO:0032259 (methylation), and GO:0005975 (carbohydrate metabolic process). The CC (cellular component) includes GO:0022627 (cytosolic small ribosomal subunit), GO:0015935 (small ribosomal subunit), GO:0022625 (cytosolic large ribosomal subunit), GO:0005840 (ribosome), and GO:0016021 (integral component of membrane). The MF (molecular function) includes GO:0005199 (structural constituent of cell wall), GO:0004622 (lysophospholipase activity), GO:0004356 (glutamate-ammonia ligase activity), GO:0004096 (catalase activity), GO:0005375 (copper ion transmembrane transporter activity), GO:0004100 (chitin synthase activity), GO:0008131 (primary amine oxidase activity), GO:0003735 (structural constituent of ribosome), GO:0004190 (aspartic-type endopeptidase activity), GO:0005507 (copper ion binding), GO:0016614 (oxidoreductase activity, acting on CH-OH group of donors), GO:0008080 (N-acetyltransferase activity), and GO:0008168 (methyltransferase activity). (E) Top 10 enrichment KEGG pathways of the DEGs. (F) qRT-PCR verification of the expression levels of 10 selected genes in the WT and Δ*Cfgcn5* mutant of *C. fructicola* in association with the transcriptome data. (G) Transcriptional expression levels of autophagy related genes in the WT and Δ*Cfgcn5*. Error bars represent the standard deviation with 3 three replicates. Asterisks indicate statistically significant differences (*, *P* < 0.05; **, *P* < 0.01).

To explore the regulatory mechanism of CfGcn5, a transcriptome analysis was applied for the wild-type (WT) and Δ*Cfgcn5* mutant by RNA sequencing (RNA-seq). Three biological replicates were established for each strain, and more than 90% of the reads in the 6 RNA-seq data sets were mapped to the genome of *C. fructicola* (GenBank assembly accession number: GCA_000319635.2). A principal component analysis (PCA) showed significant separation between the WT and Δ*Cfgcn5* mutant, with samples clustered among biological replicates (Fig. 1A). A gene expression analysis revealed that 9,693 genes were expressed in both the WT and the Δ*Cfgcn5* mutant, and 869 and 953 genes were uniquely detected in the WT and the Δ*Cfgcn5* mutant, respectively (Fig. 1B). A differentially expressed genes (DEGs) analysis revealed that 1,808 genes were upregulated and 2,581 genes were downregulated by at least 2-fold (*P* < 0.01) in the Δ*Cfgcn5* mutant (Fig. 1C).

All of the DEGs were further analyzed by gene ontology (GO) enrichment (*P* < 0.01), and 28 terms were enriched in total across the biological process (10 terms), cellular component (5 terms), and molecular function (13 terms) (Fig. 1D). These 28 terms were mainly clustered into 7 groups: ribosome, catalytic and metabolic process, copper ion homeostasis, cell wall and membrane, oxidoreductase activity, N-acetyltransferase and methyltransferase activity, and other enzymatic activity. The top 10 enriched KEGG pathways of the DEGs were also analyzed. Apart from the ribosome and steroid biosynthesis, the other 8 pathways were all related to metabolism, especially to primary metabolism (Fig. 1E).

To confirm the gene-expression patterns, five upregulated and five downregulated genes were selected randomly. The expression patterns of these genes were analyzed by quantitative real-time polymerase chain reaction (qRT-PCR), and all were consistent with those from the transcriptome data (Fig. 1F). In the RNA-seq data, several autophagy-related genes were among the DEGs, and we focused on the 15 orthologs of the pathogenicity-related *ATG* genes of M. oryzae (*P* < 0.05) (Table S1). Further analyses using qRT-PCR validated that *CfATG2*, *CfATG8*, and *CfATG15* were significantly downregulated, while *CfATG3*, *CfATG4*, *CfATG6*, *CfATG9*, *CfATG10*, *CfATG12*, *CfATG13*, and *CfATG18* were significant upregulated in the Δ*Cfgcn5* mutant (Fig. 1G) compared with the WT. These results indicated that CfGcn5 regulates the expression of multiple genes, including the autophagy-related genes.

10.1128/mbio.01956-22.6 TABLE S1 The expression levels of *CfATG* genes in transcriptome data. Download Table S1, DOC file, 0.01 MB. Copyright © 2022 Zhang et al. 2022 Zhang et al. https://creativecommons.org/licenses/by/4.0/ This content is distributed under the terms of the Creative Commons Attribution 4.0 International license.

### CfGcn5-dependent acetylation and phosphorylation are important for the response to rapamycin stress and pathogenicity.

**FIG 2:**
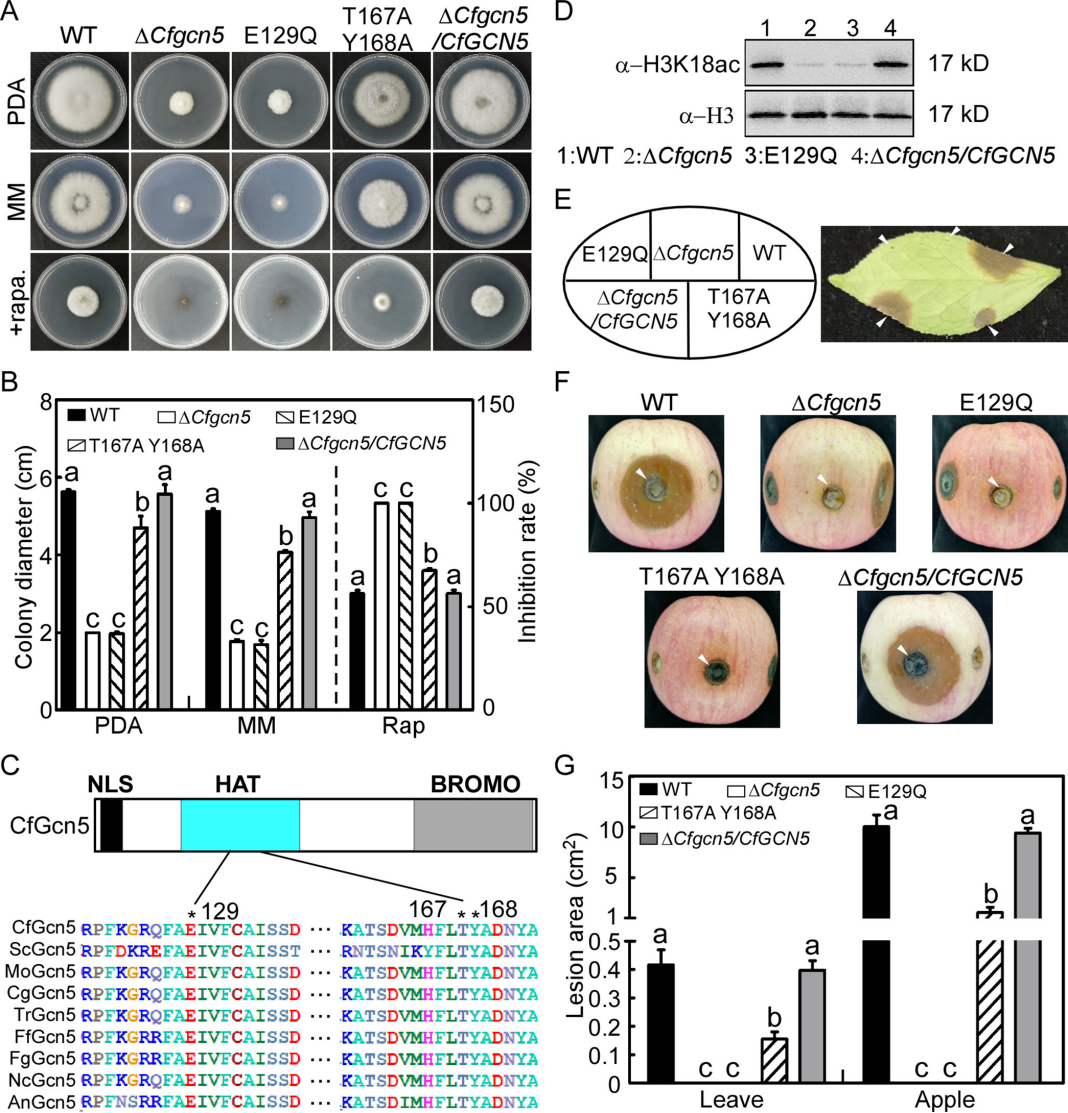
CfGcn5-dependent acetylation and phosphorylation are important for the response to rapamycin stress and pathogenicity. (A) WT, Δ*Cfgcn5* mutant, point-mutated strains E192Q and T167AY168A, and complemented strain Δ*Cfgcn5/CfGCN5* were cultured in PDA, MM, or PDA with 25 nM rapamycin for 4 d. (B) Statistical analysis of the colony diameter of the strains in PDA and MM and the inhibition rates of the strains to rapamycin stress. Error bars indicate the standard deviation with three replicates, and different letters represent statistically significant differences (*P* < 0.01). (C) The structure and domain prediction of CfGcn5. The asterisk indicates the conserved glutamic acid, threonine, and tyrosine residues of Gcn5 proteins among fungi. The related species names are as follows: *C. fructicola*, Saccharomyces cerevisiae, M. oryzae, *C. gloeosporioides*, Trichoderma reesei, Fusarium fujikuroi, F. graminearum, Neurospora crassa, and A. nidulans. (D) Immunoblot analysis of the H3K18 acetylation with α-H3K18ac and α-H3 primary antibodies. (E) *C. oleifera* leaves were inoculated with mycelial plugs of the related strains and photographed at 4 days post incubation (dpi). Arrows indicate the inoculated areas. (F) The apples were inoculated with mycelial plugs of the related strains and photographed at 5 dpi. Arrows indicate the inoculated areas. (G) Statistical analysis of lesion areas on *C. oleifera* leaves and apples. Error bars represent the standard deviation with three replicates, and different letters represent statistically significant differences (*P* < 0.01).

To test whether CfGcn5 regulates autophagy, we examined the sensitivity of various strains to rapamycin, which induces autophagy via the target of rapamycin (TOR) by phosphorylating the core Atg proteins (38). We found that the Δ*Cfgcn5* mutant showed significantly higher inhibition rates than did the WT and complemented strain (Fig. 2A and B). We previously demonstrated that CfGcn5 contained conserved NLS, HAT, and BROMO domains and that HAT acted as the most important domain (7). The H3K18 was the reported acetylated lysine residue of Gcn5, and the enzymatically inactive of Gcn5 would dismiss its HAT activity (39–41). To further investigate the roles of the HAT domain in the response to rapamycin stress, we obtained an enzymatically inactive strain of CfGcn5, which changed the conserved enzymatic residue E129 (glutamic acid) (Fig. 2C) to Q (glutamine). As expected, we found that the H3K18ac levels of the E129Q strain and Δ*Cfgcn5* mutant were significantly decreased compared to that of the WT and complemented strain (Fig. 2D). Meanwhile, the T167 (threonine) and Y168 (tyrosine) residues in the HAT domain are highly conserved in Gcn5 homologs (Fig. 2C), which are phosphorylated by Snf1 for regulating transcription in S. cerevisiae (42). Amino acid substitution of both them to A (alanine) would hypophosphorylate Gcn5 proteins (42). We found that the E129Q mutant showed similar inhibition rates as did the Δ*Cfgcn5* mutant to rapamycin, while the T167AY168A double mutant showed moderate inhibition rates between those of the WT and Δ*Cfgcn5* mutant (Fig. 2A and B). Additionally, we found that the E129Q mutant showed the same growth rate as did the Δ*Cfgcn5* mutant, while the T167AY168A double mutant showed a moderate growth rate between those of the WT and Δ*Cfgcn5* mutant in PDA and MM medium (Fig. 2A and B). Moreover, the pathogenicity assays on *C. oleifera* leaves and apples showed that E129Q caused no lesion, similar to the Δ*Cfgcn5* mutant, whereas T167AY168A caused lesions that were significantly lesser than those caused by the WT (Fig. 2E–G). These results demonstrate that CfGcn5 regulates the response to rapamycin stress and pathogenicity and that the regulation is dependent on its acetylation and phosphorylation activities.

### CfGcn5 negatively regulates autophagy.

To investigate how CfGcn5 regulates autophagy, we identified CfAtg8, the ortholog of which acts as a reliable marker in other organisms (29, 43), for autophagic flux analysis. We constructed two fusion proteins of GFP, tagged to the C-terminal and N-terminal ends of CfAtg8, with the RP27 promoter. We found that only GFP-CfAtg8 showed punctate fluorescence, whereas CfAtg8-GFP was uniformly distributed throughout the cytoplasm (Fig. S1A). Thus, we selected GFP-CfAtg8 for further study. To test whether the punctate fluorescence indicates autophagosomes, the RFP tag was fused to CfApe1, a homolog of the yeast autophagosome marker Ape1. We found that most punctate green fluorescence was colocalized with CfApe1 (Fig. S1A), supporting the claim that CfAtg8 is localized to the autophagosomes. Additionally, both GFP-CfAtg8 and CfAtg8-GFP restored the pathogenicity defects of the Δ*Cfatg8* mutant on *C. oleifera* leaves and apples, indicating their normal function in *C. fructicola* (Fig. S1B–D).

10.1128/mbio.01956-22.2 FIG S1 CfAtg8 localizes to the autophagosomes. (A) The localization pattern of CfAtg8-GFP and GFP-CfAtg8.The arrows indicate the colocalization of GFP-CfAtg8 with CfApe1-RFP, which was expressed as the autophagosome marker. (B) The mycelial plugs of the WT, Δ*Cfatg8* mutant, and complemented strains CfAtg8-GFP and GFP-CfAtg8 were inoculated on the wounded *C. oleifera* leaves and photographed at 4 dpi. (C) The mycelial plugs of the WT, Δ*Cfatg9* mutant, and complemented strains CfAtg9-GFP and GFP-CfAtg9 were inoculated on the wounded apples and photographed at 5 dpi. The arrowheads indicate the inoculated areas. (D) Statistical analysis of lesion areas on *C. oleifera* leaves and apples. Error bars represent the standard deviation with three replicates, and asterisks represent statistically significant differences (*P* < 0.01). Bar = 5 μm. Download FIG S1, TIF file, 1.9 MB. Copyright © 2022 Zhang et al. 2022 Zhang et al. https://creativecommons.org/licenses/by/4.0/ This content is distributed under the terms of the Creative Commons Attribution 4.0 International license.

**FIG 3:**
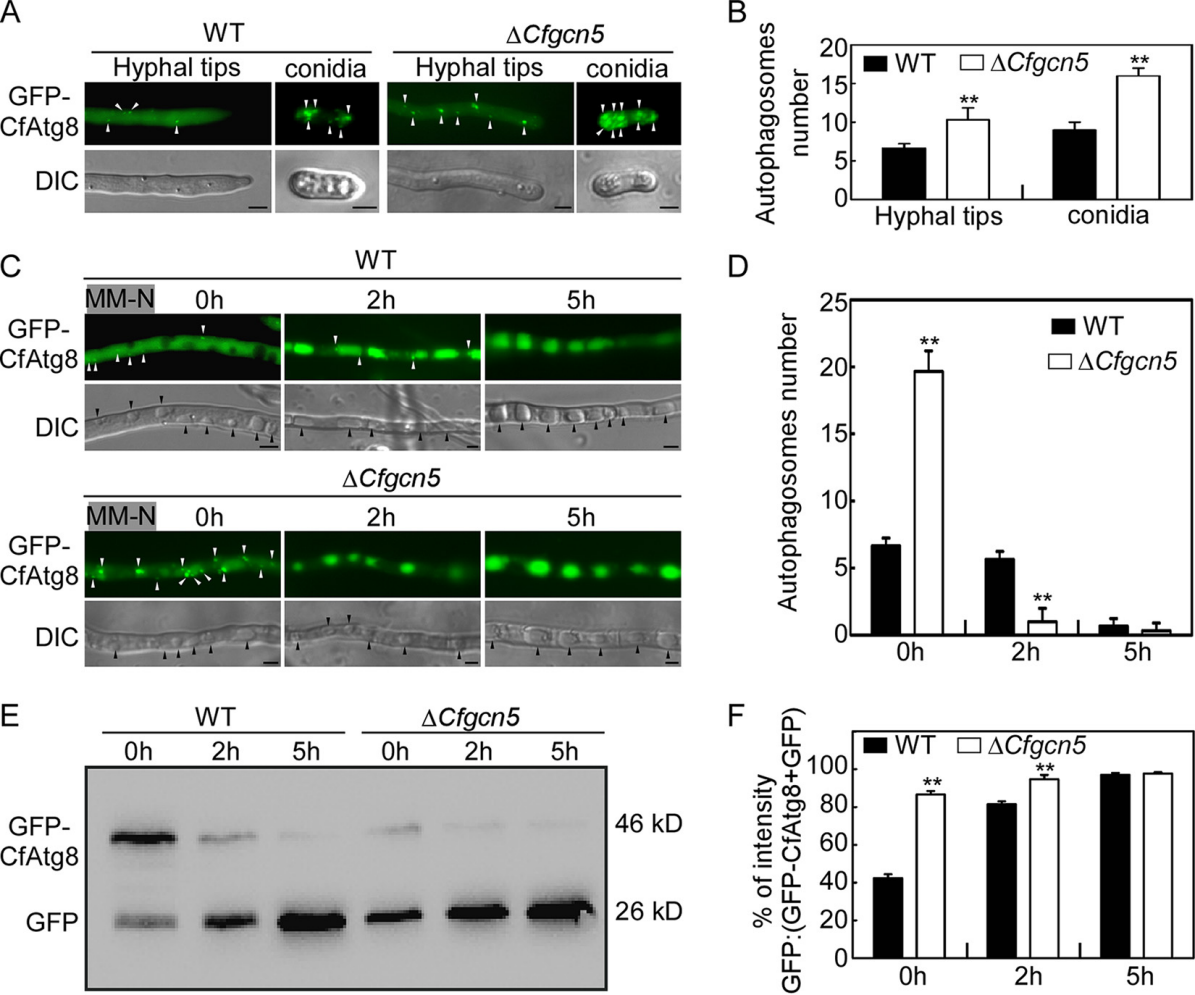
CfGcn5 negatively regulates autophagy. (A) Micrographs of GFP-CfAtg8 labeled autophagosomes in the WT and Δ*Cfgcn5* mutant. (B) Statistical analysis of autophagosome numbers in the WT and Δ*Cfgcn5* mutant. More than 50 hyphal tips (with a length of about 50 μm) and conidia were observed for the mean number. The experiments were repeated three times and yielded similar results. (C) The WT and Δ*Cfgcn5* mutant strains, transformed with GFP-MoAtg8, were incubated in MM-N for 2 h and 5 h. Then, the autophagy was observed with a microscope. (D) Statistical analysis of autophagosome numbers in the WT and Δ*Cfgcn5* mutant for MM-N induction. More than 50 hyphae were observed for the mean number. The experiments were repeated three times and yielded similar results. (E) Immunoblot analysis of GFP-CfAtg8 proteolysis. The upper and lower lanes point to the intact GFP-Atg8 (46 kDa) and free GFP (26 kDa), respectively. (F) The level of autophagy was estimated by calculating the amount of free GFP relative to the total amount of intact GFP-CfAtg8 plus free GFP. Asterisks indicate statistically significant differences (*P* < 0.01). White arrows indicate autophagosomes, and black arrows indicate vacuoles. Bar = 5 μm.

Next, we introduced the GFP-CfAtg8 into the WT and Δ*Cfgcn5* mutant and found that the Δ*Cfgcn5* mutant showed significantly more autophagosomes in the hyphal tips and conidia than did the WT (Fig. 3A and B). Nutrient deprivation (under MM-N treatment) is the other way to induce autophagy by inactivating TOR (44). Before MM-N treatment, GFP fluorescence was observed in the cytoplasm but not in the vacuoles in the WT, whereas both the cytoplasm and the vacuole could detect GFP fluorescence in the Δ*Cfgcn5* mutant, and the Δ*Cfgcn5* mutant also showed significantly more autophagosomes than did the WT in medium hypha (Fig. 3C and D). After MM-N treatment for 2 h, most of the GFP fluorescence was delivered into the vacuoles, both in the WT and Δ*Cfgcn5* mutant, but the WT still showed some cytoplasmic GFP fluorescence and significantly more autophagosomes than did the Δ*Cfgcn5* mutant. After MM-N treatment for 5 h, the vast majority of the GFP fluorescence was detected in the vacuoles, both in the WT and Δ*Cfgcn5* mutant, and the Δ*Cfgcn5* mutant showed comparable autophagosomes to those of the WT (Fig. 3C and D).

To investigate whether the change of fluorescence signals was caused by the transcriptional regulation of *CfATG8*, we examined the transcriptional abundance of *CfATG8* during autophagy in the WT and Δ*Cfgcn5* mutant. The transcription of *CfATG8* not only showed no significant difference at different time points in the same strain but also showed comparable levels between the WT and Δ*Cfgcn5* mutant when the GFP-CfAtg8 of the RP27 promoter was introduced (Fig. S2). Next, we monitored the autophagic flux via immunoblot and found that the full-length GFP-MoAtg8 (46 kDa) and free GFP (26 kDa) could be detected in both the WT and Δ*Cfgcn5* mutant. The autophagy level was then estimated by calculating the free GFP relative to the total amount of intact GFP-CfAtg8 and free GFP together. The proportion of free GFP in the Δ*Cfgcn5* mutant was significantly higher than that of the WT, following no treatment or nutrition starvation for 2 h (Fig. 3E and F), supporting the higher autophagy level in the Δ*Cfgcn5* mutant. Not until after 5 h of nutrition starvation did the Δ*Cfgcn5* mutant show a comparable proportion of free GFP to that of the WT (Fig. 3E and F). Collectively, these results indicate that CfGcn5 negatively regulates autophagy.

10.1128/mbio.01956-22.3 FIG S2 Transcriptional expression levels of the *CfATG8* gene in the WT and Δ*Cfgcn5* mutant. WT and Δ*Cfgcn5* mutant strains, transformed with GFP-CfAtg8, were incubated in MM-N induction conditions for 0, 2, and 5 h. Then, the transcriptional levels of the *CfATG8* gene were analyzed. Download FIG S2, TIF file, 0.1 MB. Copyright © 2022 Zhang et al. 2022 Zhang et al. https://creativecommons.org/licenses/by/4.0/ This content is distributed under the terms of the Creative Commons Attribution 4.0 International license.

### Induced autophagy promotes the degradation of CfGcn5.

**FIG 4:**
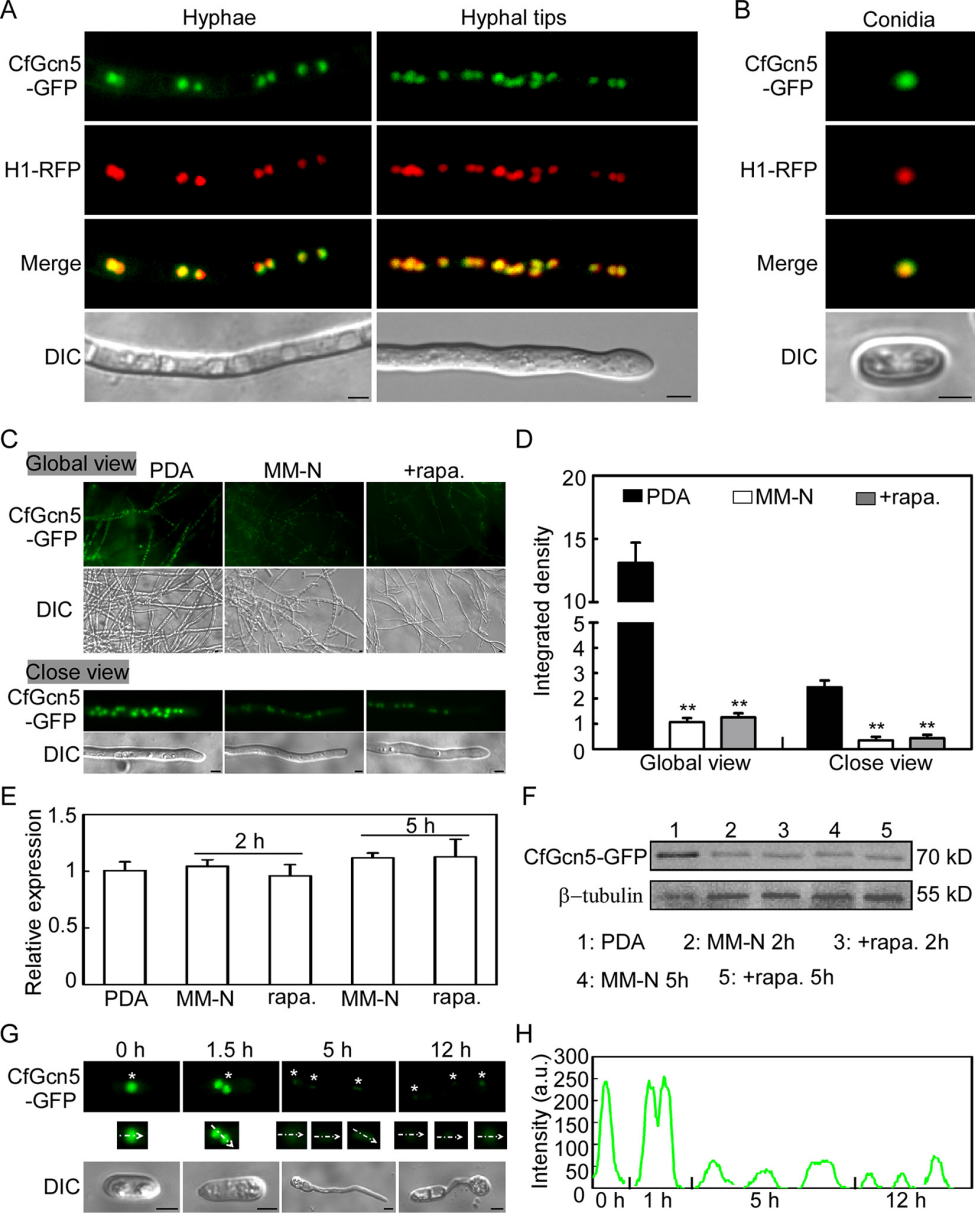
Induced autophagy promotes the degradation of CfGcn5. (A and B) H1 was used as a nucleus marker. The colocalization of CfGcn5-GFP and H1-RFP showed that CfGcn5 localized to the nucleus in hyphae and conidia. (C) The CfGcn5-GFP-expressing mycelia, the culturing in PDA, and the rapamycin/MM-N induction were examined with a global view for the mycelia and with close observation for the hyphal tips. (D) Statistical analysis of the intensity of CfGcn5-GFP, quantified with ImageJ. (E) Transcriptional expression levels of *CfGCN5* in PDA and MM-N/rapamycin induction conditions. (F) Immunoblot analysis of CfGcn5-GFP protein. (G) The localization pattern of CfGcn5 in the conidia and conidia incubated on coverslips at 1.5, 5, and 12 hpi. Asterisks indicate the CfGcn5-GFP localized area. Arrowheads point to the areas used for determinations of fluorescence intensity. (H) The fluorescence intensity profiles by line-scan analysis with ImageJ of the areas indicated with arrowheads in (E). Bar = 5 μm.

A previous study revealed that the histone acetyltransferase MoHat1 was translocated from the nucleus to the cytoplasm for the acetylation of MoAtg3 and MoAtg9 during starvation-induced autophagy in M. oryzae (33). Thus, we monitored the dynamic changes of the localization of CfGcn5 when autophagy happens. Our results revealed that CfGcn5-GFP colocalized with H1-RFP, a nucleus marker, in the mid and tip regions of the hyphae under nutrient-rich PDA conditions (Fig. 4A). We also found the colocalization of CfGcn5-GFP with H1-RFP in the conidia (Fig. 4B), supporting the nucleus localization of CfGcn5. We speculated some cytoplasm localization of CfGcn5 under MM-N or rapamycin-induced autophagy. Unexpectedly, CfGcn5 still showed nucleus localization, but the fluorescence intensity of CfGcn5-GFP was significantly reduced under MM-N or rapamycin treatment (Fig. 4C and D). To clarify how autophagy mediates the activity of CfGcn5, we first examined the transcriptional abundance of *CfGCN5* during autophagy. The results showed comparable transcriptional levels between induction and noninduction conditions (Fig. 4E). Next, the CfGcn5-GFP protein levels were investigated via immunoblot. Consistent with the fluorescence intensity results, the protein levels of CfGcn5-GFP were significantly decreased under MM-N or rapamycin-induced autophagy (Fig. 4F).

Appressorium formation was accompanied by the autophagic fungal cell death in M. oryzae (45). Thus, we monitored the localization of CfGcn5 during appressorium formation on an inductive, artificial hydrophobic surface. At 1.5 h post incubation (hpi), the conidia contained two CfGcn5-GFP nuclei, the fluorescence intensity of which was comparable with that of conidia under no induction. At 5 hpi and 12 hpi, the fluorescence intensity of CfGcn5-GFP was significantly decreased compared to that of the conidia (Fig. 4G and H). Taken together, these results indicate that CfGcn5 is degraded during induced autophagy.

### The autophagy related proteins CfAtg8 and CfAtg9 are localized to autophagosomes, and CfAtg8 regulates the response to rapamycin stress.

To examine the role of autophagy in *C. fructicola*, we further investigated the biological functions of the autophagy related proteins CfAtg8 and CfAtg9, the orthologs of which act as a reliable marker of autophagy and the only integral membrane component of the conserved Atg proteins in yeast (46, 47), respectively. Additionally, the expression levels of *CfATG*8 and *CfATG9* were significantly downregulated and upregulated, respectively, in the Δ*Cfgcn5* mutant (Fig. 1G) compared with the WT. We previously acquired *CfATG8* gene deletion mutant Δ*Cfatg8*, and thus, we further obtained 3 independent Δ*Cfatg9* mutants (Δ*Cfatg9*-7, Δ*Cfatg9*-8, and Δ*Cfatg9*-33) (Fig. S3A and B). The CfAtg8 was localized to the autophagosomes (Fig. S1A), and we also constructed two fusion proteins of CfAtg9-GFP and GFP-CfAtg9 to observe the localization of CfAtg9. CfAtg9-GFP showed a slight fluorescent signal affecting observation, and GFP-CfAtg9 was colocalized with CfApe1-RFP, indicating that CfAtg9 was also localized to the autophagosomes (Fig. S4A). Additionally, both GFP-CfAtg9 and CfAtg9-GFP recovered the pathogenicity defects of the Δ*Cfatg9* mutant on *C. oleifera* leaves and apples, confirming their normal function in *C. fructicola* (Fig. S4B–D).

10.1128/mbio.01956-22.4 FIG S3 Generation of the *CfATG9* gene deletion mutant in *C. fructicola*. (A) Schematic illustration of the *CfATG9* gene deletion strategy in the WT. (B) Analysis of the Δ*Cfatg9* mutant by PCR with primer 1 (NBF/NBR) and primer 2 (BWF/HPHR). (C) Schematic illustration of the *CfATG9* gene deletion strategy in the Δ*Cfgcn5* mutant. (D) Analysis of the Δ*Cfatg9* mutant by PCR with primer 1 (NBF/NBR) and primer 3 (BWF/G418R). M represents the marker, plus represents the positive control, minus represents the negative control, and different numbers represent different transformants of the mutants. Download FIG S3, TIF file, 0.6 MB. Copyright © 2022 Zhang et al. 2022 Zhang et al. https://creativecommons.org/licenses/by/4.0/ This content is distributed under the terms of the Creative Commons Attribution 4.0 International license.

10.1128/mbio.01956-22.5 FIG S4 CfAtg9 localizes to the autophagosomes. (A) The localization pattern of CfAtg9-GFP and GFP-CfAtg9.The arrows indicate the colocalization of GFP-CfAtg9 with CfApe1-RFP. (B) The mycelial plugs of the WT, Δ*Cfatg9* mutant, and complemented strains CfAtg9-GFP and GFP-CfAtg9 were inoculated on the wounded *C. oleifera* leaves and photographed at 4 dpi. (C) The mycelial plugs of the WT, Δ*Cfatg9* mutant, and complemented strains CfAtg9-GFP and GFP-CfAtg9 were inoculated on the wounded apples and photographed at 5 dpi. The arrowheads indicate the inoculated areas. (D) Statistical analysis of lesion areas on *C. oleifera* leaves and apples. Error bars represent the standard deviation with three replicates, and asterisks represent statistically significant differences (*P* < 0.01). Bar = 5 μm. Download FIG S4, TIF file, 1.9 MB. Copyright © 2022 Zhang et al. 2022 Zhang et al. https://creativecommons.org/licenses/by/4.0/ This content is distributed under the terms of the Creative Commons Attribution 4.0 International license.

**FIG 5:**
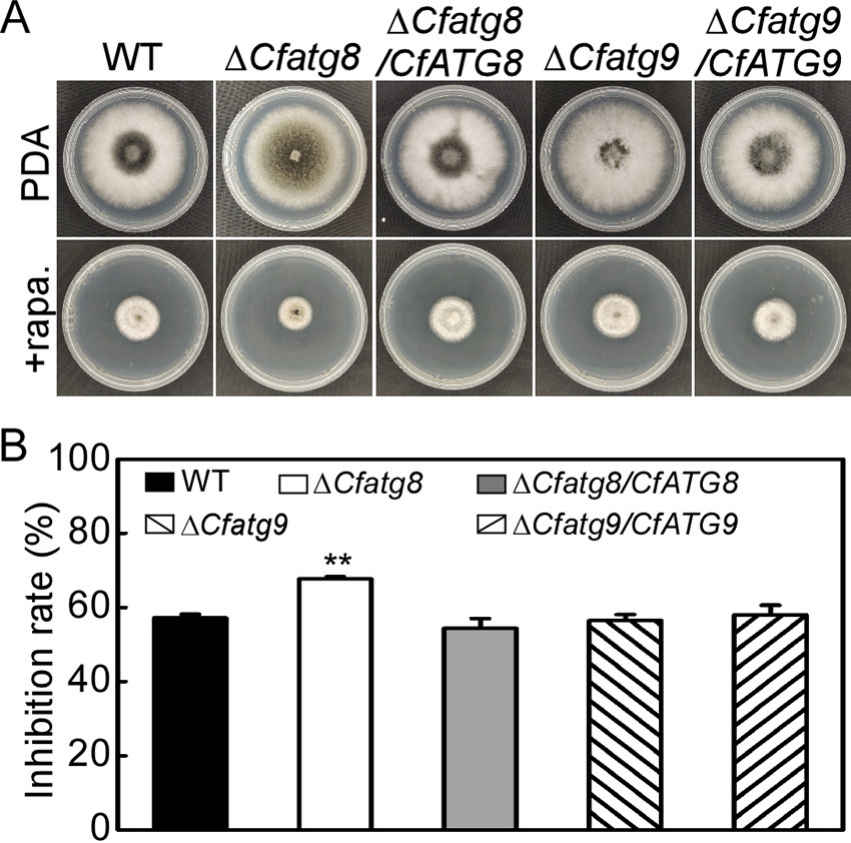
The autophagy related protein CfAtg8, but not CfAtg9, regulates the response to rapamycin stress. (A) WT, Δ*Cfatg8* mutant, Δ*Cfatg9* mutant, and complemented strains Δ*Cfatg8/CfATG8* and Δ*Cfatg9/CfATG9* were cultured in either PDA or PDA with 25 nM rapamycin for 4 d. (B) Statistical analysis of the inhibition rates of the strains to rapamycin stress. Error bars indicate the standard deviation with three replicates, and asterisks represent statistically significant differences (*P* < 0.01).

Next, we tested their roles in the response to rapamycin stress, and the results showed that the Δ*Cfatg8* mutant exhibits significantly higher inhibition rates than do the WT and complemented strain Δ*Cfatg8/CfATG8*, whereas the Δ*Cfatg9* mutant exhibited comparable inhibition rates to the WT and complemented strain Δ*Cfatg9/CfATG9* (Fig. 5A and B). This result reveals that CfAtg8, but not CfAtg9, regulates the response to rapamycin stress.

### CfAtg8 and CfAtg9 are important for pathogenicity.

**FIG 6:**
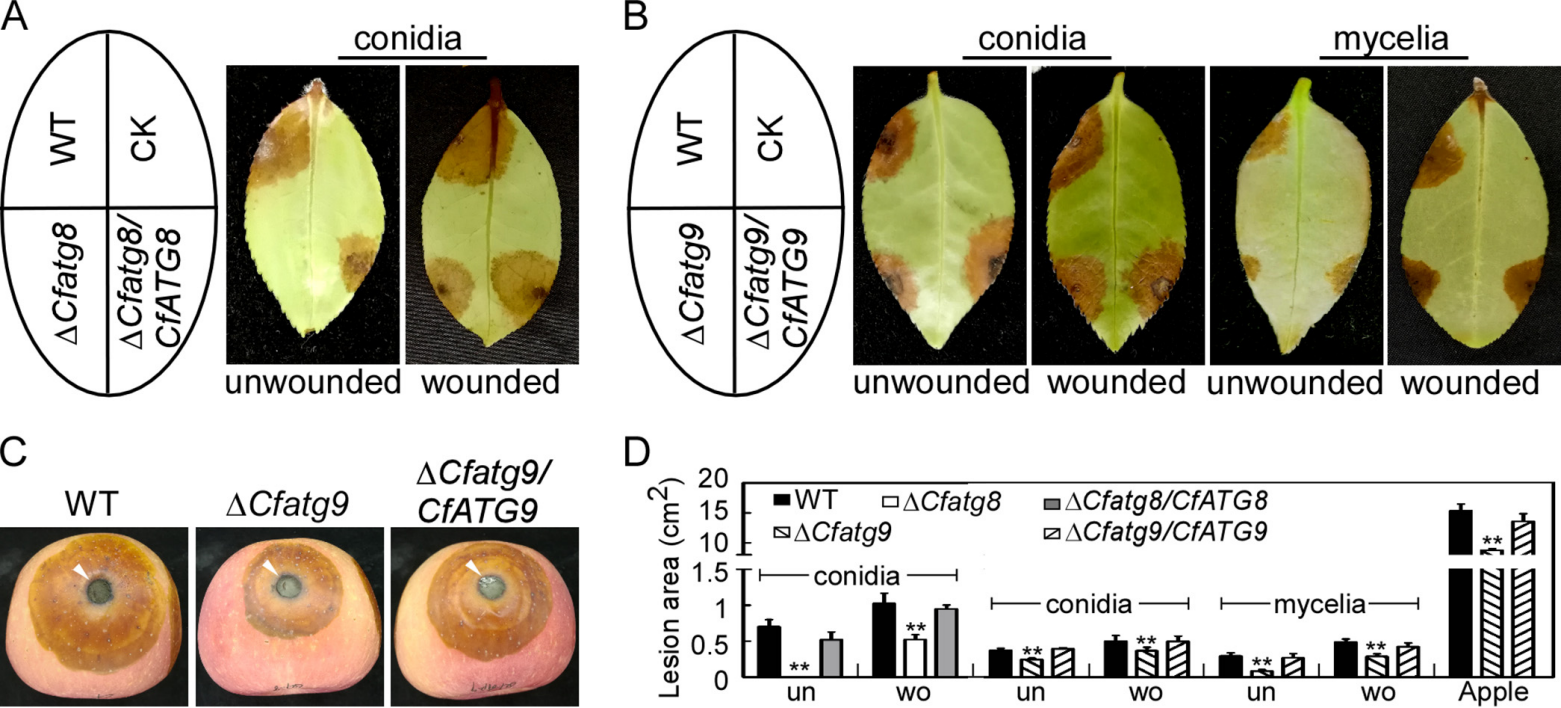
CfAtg8 and CfAtg9 are important for pathogenicity. (A) The conidial suspensions (3 × 10^5^ conidia/mL) of WT, Δ*Cfatg8*, and Δ*Cfatg8/CfATG8* were inoculated on the unwounded and wounded *C. oleifera* leaves and photographed at 4 dpi. (B) The conidial suspensions (3 × 10^5^ conidia/mL) or mycelia of WT, Δ*Cfatg9*, and Δ*Cfatg9/CfATG9* were inoculated on the unwounded and wounded *C. oleifera* leaves and photographed at 4 dpi. (C) The mycelia of WT, Δ*Cfatg9*, and Δ*Cfatg9/CfATG9* were inoculated on the wounded apples and photographed at 7 dpi. The arrowheads indicate the inoculated areas. (D) Statistical analysis of lesion areas on *C. oleifera* leaves and apples. Error bars represent the standard deviation with three replicates, and asterisks represent statistically significant differences (*P* < 0.01).

As this fungus is plant-pathogenic, we focused our interests on the contributions of CfAtg8 and CfAtg9 to pathogenicity in *C. fructicola*. When dripping the same amounts of conidial suspensions, the Δ*Cfatg8* mutant showed no lesion compared with the typical and large lesions caused by the WT and the complemented strain on healthy *C. oleifera* leaves. On wounded *C. oleifera* leaves, the Δ*Cfatg8* mutant showed some lesions, but they were still fewer than those of the WT and Δ*Cfatg8/CfATG8* (Fig. 6A and D). A similar pathogenicity defect of the Δ*Cfatg8* mutant was also observed in our previous study using mycelia for inoculation on *C. oleifera* leaves and apples. The conidial suspension and mycelia of the Δ*Cfatg9* mutant were also inoculated into *C. oleifera* leaves and apples, and the results showed that the Δ*Cfatg9* mutant exhibited lower virulence than did the WT and Δ*Cfatg9/CfATG9* in all pathogenicity tests (Fig. 6B–D).

### CfAtg8 and CfAtg9 are involved in appressorium formation and turgor pressure.

**FIG 7:**
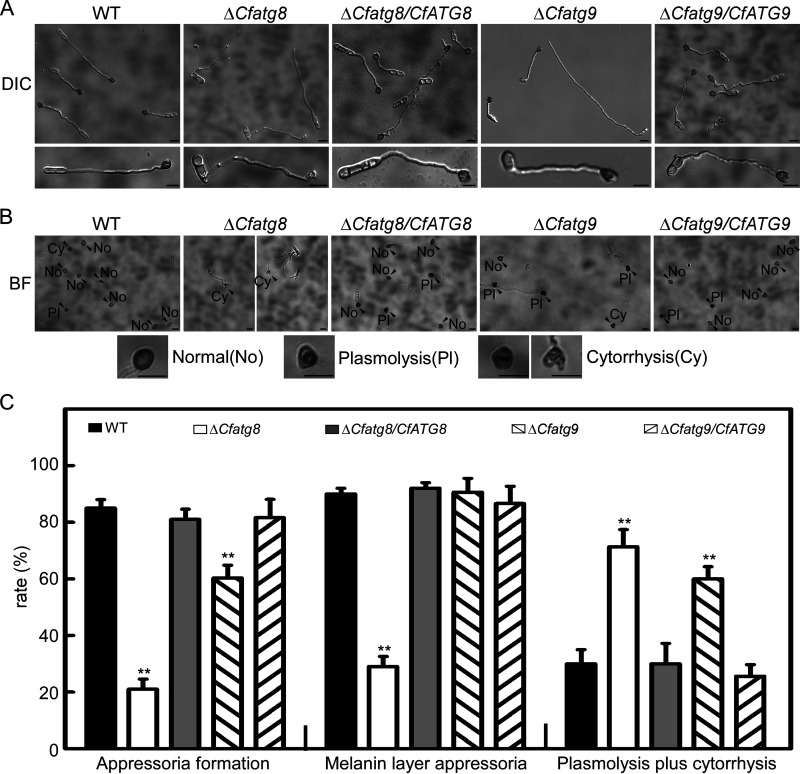
CfAtg8 and CfAtg9 are involved in appressoria formation and turgor pressure. (A) The conidial suspensions (3 × 10^5^ conidia/mL) of WT, Δ*Cfatg8*, Δ*Cfatg8/CfATG8*, Δ*Cfatg9*, and Δ*Cfatg9/CfATG9* were incubated on artificial hydrophobic surfaces. The appressoria were observed after 24 h of incubation. (B) The appressoria of the related strains after incubation in 4 M glycerol solution for 10 min. No, normal; Pl, plasmolysis; Cy, cytorrhysis. (C) Statistical analysis of appressoria formation and melanin layer appressoria rates as well as the rate of appressoria exhibiting plasmolysis and cytorrhysis. The experiments were repeated three times with three replicates, and more than 100 conidia were observed for each biological replicate. Error bars represent the standard deviation with three replicates, and asterisks represent statistically significant differences (*P* < 0.01). Bar = 10 μm.

To clarify the underlying mechanism of the pathogenicity defects of the Δ*Cfatg8* and Δ*Cfatg9* mutants, we tested their appressoria formation ability on the hydrophobic surfaces. We found that Δ*Cfatg8* and Δ*Cfatg9* showed about 20% and 60% appressoria formation rates, respectively, compared with rates of greater than 80% in the WT and complemented strains (Fig. 7A and C). Additionally, we found that less than 25% of the appressoria of Δ*Cfatg8* showed a melanin layer, which is a virulence characteristic in phytopathogenic fungi (48, 49), compared with more than 80% of the appressoria in the WT and complemented strain (Fig. 7A and C). Since strong internal turgor pressure determines the appressorium-mediated host penetration, we tested appressorial turgor via a cytorrhysis assay (50). In 4 M glycerol, 73% and 61% of appressoria showed cytorrhysis and plasmolysis in Δ*Cfatg8* and Δ*Cfatg9*, respectively, compared with about 25% in the WT and complemented strains (Fig. 7A–C). Collectively, these results indicate that CfAtg8 and CfAtg9 regulate appressorium formation and turgor pressure.

### CfAtg8 and CfAtg9 regulate mitosis during appressoria formation.

**FIG 8:**
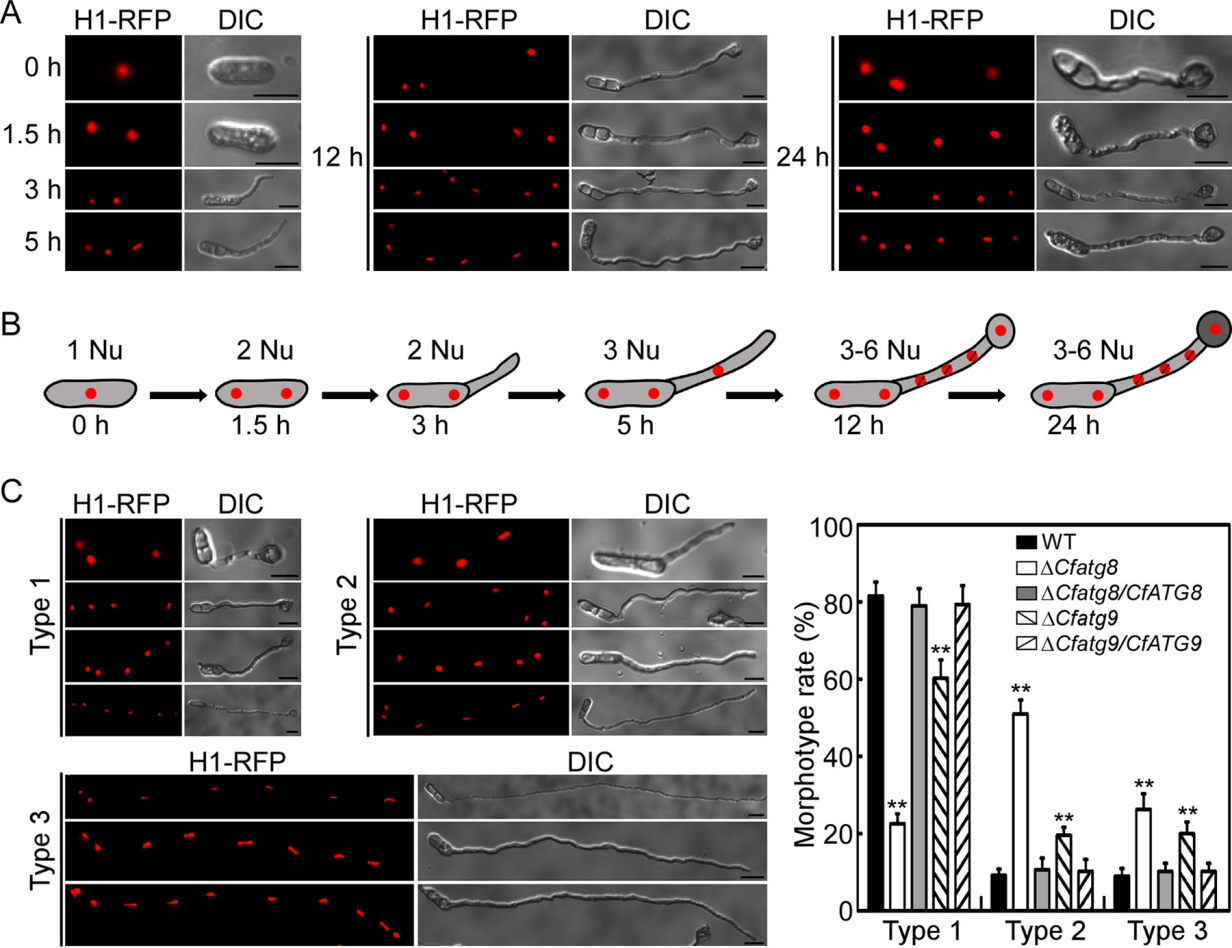
CfAtg8 and CfAtg9 regulate mitosis during the appressoria formation. (A) The conidia of WT expressing H1-RFP were incubated on artificial hydrophobic surfaces and were observed by epifluorescence microscopy at 0, 1.5, 3, 5, 12, and 24 h. (B) Model of the changes of nucleic numbers during the appressoria formation. (C) Detailed observation and statistics for the conidia of WT, Δ*Cfatg8*, and Δ*Cfatg9*, expressing H1-RFP, incubated on artificial hydrophobic surfaces at 24 h. The morphotype rate of the germinated conidia were rated from Type 1 to 3: Type 1, the appressoria with 3 to 6 nuclei; Type 2, the germ tubes with 3 to 6 nuclei; and Type 3, the germ tubes with more than 7 nuclei. Error bars represent the standard deviation with three replicates, and asterisks represent statistically significant differences (*P* < 0.01). Bar = 10 μm.

To explore the possible cause of the appressoria formation defect, we observed mitosis, which is essential for appressoria formation in *Colletotrichum* and *Magnaporthe* (45, 51, 52). The H1-RFP was introduced into the WT, Δ*Cfatg8*, Δ*Cfatg9*, and complemented strains for live-cell imaging, and their conidia were incubated on the inductive hydrophobic surface. The conidia of the WT contained one nucleus in each conidia, and mitosis was first observed in conidia at 1.5 hpi. Then, the germ tubes emerged at 3 hpi, followed by the second mitosis and the nucleus movement to the germ tubes at 5 hpi. At 12 hpi, the germ tubes underwent 0 to 3 mitoses, and one nucleus moved to the differentiated incipient appressorium. At 24 hpi, mature appressoria formed, and the germinated conidia still carried 3 to 6 nuclei (Fig. 8A). Based on these results, we established a model of mitosis during the appressoria formation in *C. fructicola* (Fig. 8B).

Next, we observed mitosis during appressorium formation in the Δ*Cfatg8* and Δ*Cfatg9* mutants. We found that the germinated conidia of both the Δ*Cfatg8* and the Δ*Cfatg9* mutants carried at least 2 nuclei, similar to the WT, supporting that all of them could enter into mitosis (Fig. 8C and D). Interestingly, we found that some of the expanded germ tubes of the Δ*Cfatg8* and Δ*Cfatg9* mutants contained more than 7 nuclei. Thus, we classified the germinated conidia to three types: Type 1, the appressoria with 3 to 6 nuclei; Type 2, the germ tubes with 3 to 6 nuclei; and Type 3, the germ tubes with more than 7 nuclei. We found that the rates of Type 2 and Type 3 in the Δ*Cfatg8* and Δ*Cfatg9* mutants were significantly higher than those observed in the WT and complemented strains (Fig. 8C and D). These results demonstrated accelerated mitosis in the Δ*Cfatg8* and Δ*Cfatg9* mutants, supporting the roles of CfAtg8 and CfAtg9 in mitosis regulation.

### CfAtg9 positively regulates autophagy.

**FIG 9:**
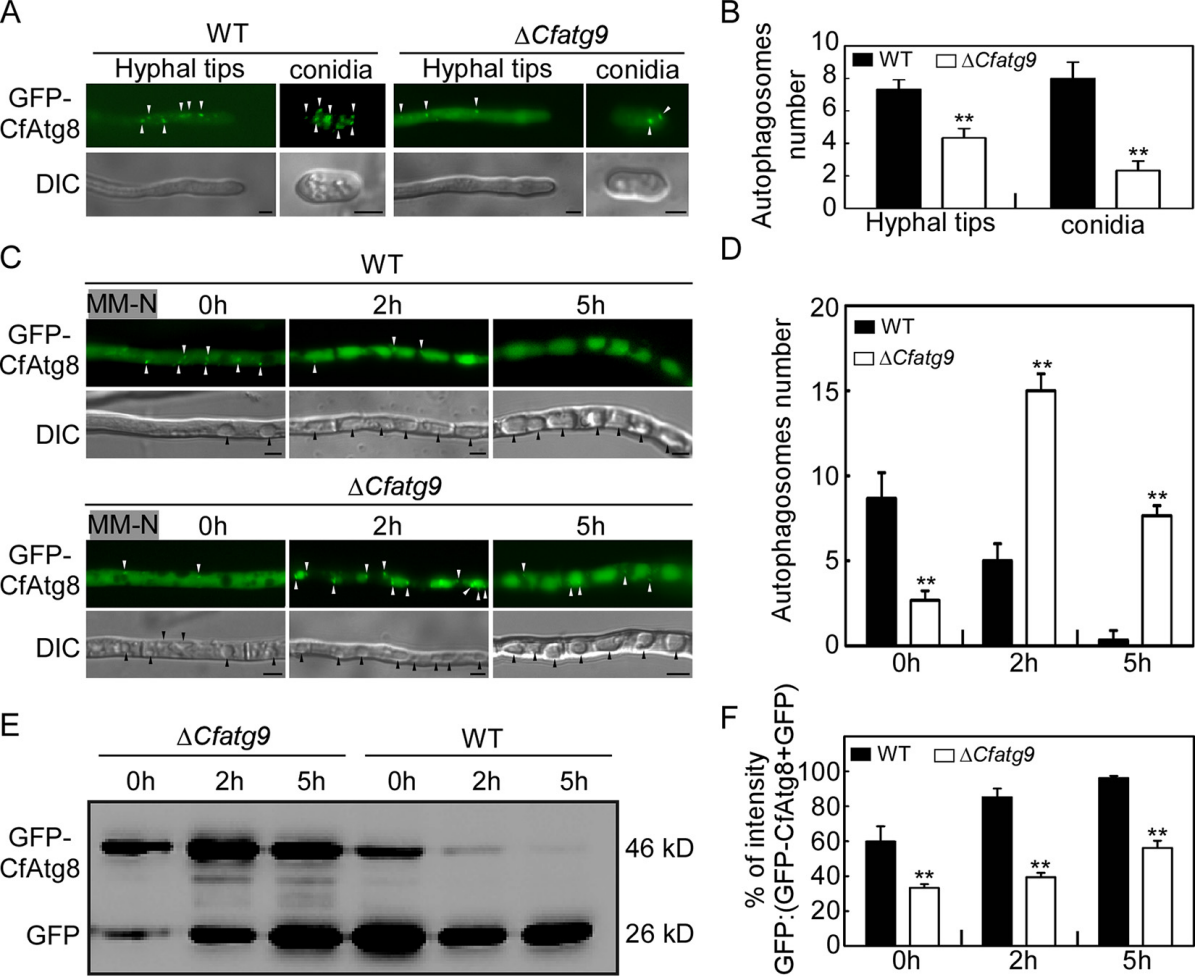
CfAtg9 positively regulates autophagy. (A) Micrographs of GFP-CfAtg8 labeled autophagosomes in the WT and Δ*Cfatg9* mutant. (B) Statistical analysis of autophagosome numbers in the WT and Δ*Cfatg9* mutant. More than 50 hyphal tips (with a length of about 50 μm) and conidia were observed for the mean number. The experiments were repeated three times and yielded similar results. (C) The WT and Δ*Cfatg9* mutant strains, transformed with GFP-MoAtg8, were incubated in MM-N for 2 h and 5 h. Then, the autophagy was observed with a microscope. (D) Statistical analysis of autophagosome numbers in the WT and Δ*Cfatg*9 mutant for MM-N induction. More than 50 hyphae were observed for the mean number. The experiments were repeated three times and yielded similar results. (E) Immunoblot analysis of GFP-CfAtg8 proteolysis. The upper and lower lanes point to the intact GFP-Atg8 (46kDa) and free GFP (26kDa), respectively. (F) The level of autophagy was estimated by calculating the amount of free GFP relative to the total amount of intact GFP-CfAtg8 plus free GFP. Asterisks indicate statistically significant differences (*P* < 0.01). White arrows indicate autophagosomes, and black arrows indicate vacuoles. Bar = 5 μm.

To explore the possible cause of the defect on turgor pressure, we detected the autophagy level, which is essential for appressoria turgor generation in M. oryzae (53). We found that the Δ*Cfatg9* mutant showed significantly fewer autophagosomes than did the WT in the hyphal tips and conidia (Fig. 9A and B). Before MM-N treatment, GFP fluorescence was observed in the cytoplasm, but not in the vacuoles, in the middle hyphae of both the WT and the Δ*Cfatg9* mutant. However, the Δ*Cfatg9* mutant showed significantly fewer autophagosomes than did the WT. Upon MM-N treatment for 2 h and 5 h, most of the GFP fluorescence was delivered into the vacuole, both in the WT and the Δ*Cfatg9* mutant, but the Δ*Cfatg9* mutant showed significantly more autophagosomes surrounding the vacuoles than did the WT (Fig. 9C and D). The autophagy level was further estimated via immunoblot. The relative ratio of free GFP in the Δ*Cfatg9* mutant was observed to be significantly lower than that of the WT under noninduction and following 2 h or 5 h of MM-N treatment (Fig. 9E and F), supporting the reduced autophagy level in the Δ*Cfatg9* mutant. Collectively, these results indicate that CfAtg9 positively regulates autophagy.

### The degradation of CfGcn5 is not dependent on CfAtg9-regulated autophagy.

**FIG 10:**
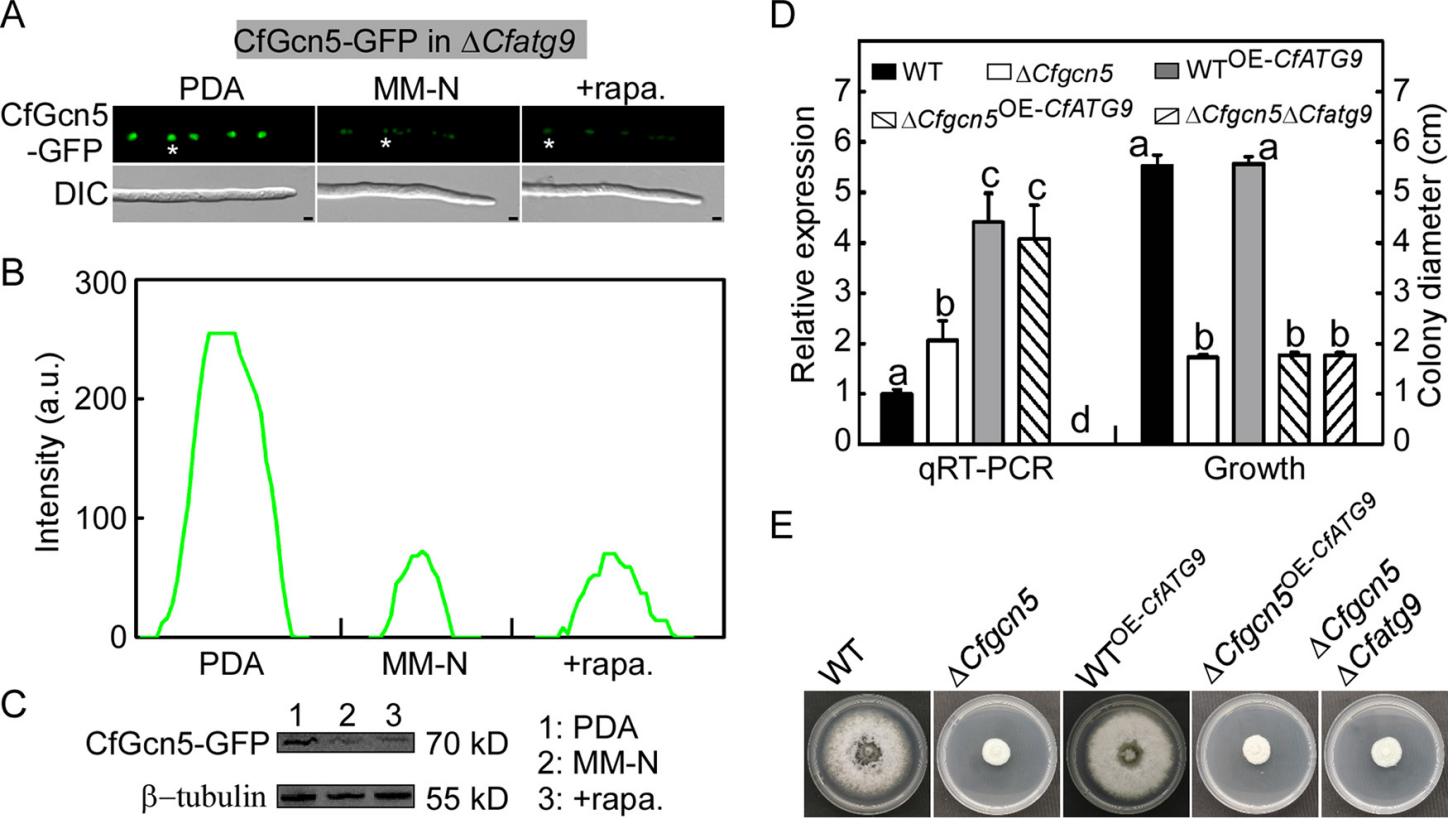
The degradation of CfGcn5 is not dependent on the CfAtg9 regulated autophagy. (A) The hyphal tips of CfGcn5-GFP expressed in the Δ*Cfatg9* mutant were observed under PDA and MM-N/rapamycin induction. (B) The fluorescence intensity profiles by line-scan analysis with ImageJ of the area indicated with asterisks in (A). Bar = 5 μm. (C) Immunoblot analysis of the CfGcn5-GFP protein. (D) Transcriptional expression levels of the *CfATG9* gene and a statistical analysis of the colony diameter of the WT, Δ*Cfgcn5*, WT^OE-^*^CfATG9^*, Δ*Cfgcn5*^OE-^*^CfATG9^*, and Δ*Cfgcn5*Δ*Cfatg9* strains. Different letters represent statistically significant differences. (E) The strains were cultured on PDA plates for 4 d and then photographed.

To test whether CfAtg9-regulated autophagy could feedback-mediate the degradation of CfGcn5, we introduced the CfGcn5-GFP into the Δ*Cfatg9* mutant. A microscopic observation and an immunoblot analysis revealed that CfGcn5-GFP was still degraded in the Δ*Cfatg9* mutant during autophagy (Fig. 10A–C), supporting the claim that the degradation of CfGcn5 is not dependent on CfAtg9-regulated autophagy. As the transcriptional level of *CfATG9* was significantly reduced in the Δ*Cfgcn5* mutant (Fig. 1G), to examine whether *CfGCN5* and *CfATG9* have any genetic interaction, we overexpressed *CfATG9* with the RP27 promoter in the WT (WT^OE-^*^CfATG9^*) and Δ*Cfgcn5* mutant (Δ*Cfgcn5*^OE-^*^CfATG9^*). The transcriptional abundance of *CfATG9* in the WT^OE-^*^CfATG9^* and Δ*Cfgcn5*^OE-^*^CfATG9^* were 4.4-fold and 4.1-fold, respectively, relative to that of the WT (Fig. 10D). Additionally, we further deleted the *CfATG9* gene in the Δ*Cfgcn5* mutant (Δ*Cfgcn5*Δ*Cfatg9*) (Fig. S3C and D). We found that the WT^OE-^*^CfATG9^* showed no significant difference from the WT in growth and that the colony diameters of Δ*Cfgcn5*, Δ*Cfgcn5*^OE-^*^CfATG9^*, and Δ*Cfgcn5*Δ*Cfatg9* were also generally comparable (Fig. 10D and E).

## DISCUSSION

Acetylation and deacetylation of histone and nonhistone proteins are critical epigenetic mechanisms of gene regulation and protein function (54, 55). In the past 10 years, the importance of acetylation in plant fungal pathogenesis has been broadly appreciated (33, 56, 57). We previously revealed that the histone acetyltransferase CfGcn5 regulates growth, conidiation, and pathogenicity in *C. fructicola* and that the nucleus localization of CfGcn5 is essential for its full function (7). Here, we not only showed that CfGcn5 regulates global gene expression but also provided evidence to reveal that CfGcn5 was an autophagy repressor that undergoes degradation to regulate autophagy-dependent pathogenicity in *C. fructicola*.

Generally, histone acetylation has positive roles in transcription, whereas deacetylation is related to transcriptional inactivation (58). The much higher number of downregulated genes (2,581) than that of upregulated genes (1,808) and the low acetylation level of H3K18 in the Δ*Cfgcn5* mutant indicated that CfGcn5 positively regulates gene expression through histone acetylation, which is also consistent with the results of the transcriptome analysis of FgGcn5 in F. graminearum (18). The cell wall organization, structural constituent of the cell wall, chitin synthase activity, response to oxidative stress, and hydrogen peroxide catabolic process of 28 enrichment GO terms might explain the previously found defects of the Δ*Cfgcn5* mutant in its responses to cell wall stress, oxidative stress, and the chitin distribution. Further analysis of KEGG pathways suggested that CfGcn5 mainly regulates the ribosome and primary metabolism. However, this is contrary with its critical roles in secondary metabolism in A. nidulans under bacteria accompaniment (59, 60). One reason for this is that most secondary metabolism genes are silent under normal conditions or in hyphal stages, which is also supported by the studies in A. nidulans (17, 61).

In *Drosophila*, Gcn5 regulates the expression of autophagy-related genes by acetylating their transcription factor TFEB (62). The different expression levels of multiple *ATG* genes in the Δ*Cfgcn5* mutant might foretell the importance of CfGcn5 in autophagy, which is further highlighted by its defects on rapamycin stress. Additionally, we indicated that HAT acts as the most important domain of CfgCcn5 in our recent work (7). Here, we further concluded that the acetylated residue E129 in the HAT domain is the most important amino acid, as the E129Q-specific mutant strain exhibited a comparably low acetylation level and similar phenotypic defects to those of the Δ*Cfgcn5* mutant. Meanwhile, the defects of the T167AY168A-specific mutant showed that the phosphorylation of CfGcn5 by Snf1, its homolog, is also essential for the pathogenicity of *C. fructicola*, as described in our previous study (63). Therefore, it might also be important for its roles in autophagy and pathogenicity.

Autophagy is a conserved and critical process for eukaryotic cells, and Atg8 is the most pivotal and reliable marker for autophagic flux analysis (27, 64, 65). Deletion of the *CfGcn5* gene resulted in accumulated numbers of autophagosomes in the hyphal tips and conidia and induced basal autophagy under normal conditions, indicating the inhibitory role of CfGcn5 in autophagy. This result was further supported by the higher levels of autophagic flux in the Δ*Cfgcn5* mutant than in the WT under MM-N induced autophagy. Our findings are consistent with the studies in M. oryzae, *Drosophila*, and humans and support the conserved roles of the Gcn5 protein in repressing autophagy (34, 62). This raises the issue of how to eliminate or weaken the above-mentioned repressed state during autophagy for *C. fructicola*. Another HAT protein, MoHat1, could shuttle between the nucleus and the cytoplasm to regulate autophagy in M. oryzae (33). Unexpectedly, CfGcn5 did not translocate from the nucleus to the cytoplasm, but it did show degradation under MM-N or rapamycin-induced autophagy. Appressoria formation was accompanied by autophagy (45, 66, 67). As CfGcn5 is degraded under autophagy, we hypothesized that this might also be happening during appressoria formation. Actually, the significantly decreased level of CfGcn5 during appressoria formation further supports the degradation of CfGcn5 under autophagy. These results were in agreement with a recent study on the nonappressoria-forming pathogen F. graminearum, which also showed that autophagy stimulates the degradation of fungal FgGcn5 (35). Collectively, CfGcn5 negatively regulates autophagy, and it undergoes degradation when autophagy is induced.

Despite the functions of autophagy in the pathogenicity of fungi already being highlighted (31, 68), its roles in *Colletotrichum* spp. remain largely unknown. The *CoATG26* is essential for pathogenicity in *Colletotrichum orbiculare* (69), but *MoATG26* is not involved in pathogenicity in M. oryzae (29), indicating that the functions of autophagy in phytopathogenicity might be species-dependent. Again, although both *CoATG8* and *MoATG8* deletion mutants showed no pathogenicity in *C. orbiculare* or in M. oryzae, these losses of virulence were due to different causes. The Δ*Coatg8* mutant was impaired in normal appressoria formation, and the Δ*Moatg8* mutant showed defects on the conidial cell death of appressoria (45, 69). This raises the issue of how autophagy functions in *C. fructicola*. Our results revealed that Δ*Moatg8* and Δ*Moatg9* mutants had defects on pathogenicity that were caused by two direct underlying mechanisms. First, the appressoria formation rates of both mutants were significantly decreased, partially due to the defects on mitosis, an essential process for appressoria formation in *Colletotrichum* and *Magnaporthe* (45, 51, 52). Second, the turgor pressure of both mutants was also reduced, likely due to the defects on autophagy, which is essential for appressoria turgor generation in M. oryzae (53). Thus, we found that CfAtg8 is involved not only in appressoria formation but also in appressoria turgor pressure, and this might take over both of the roles of Atg8 proteins in *C. orbiculare* and M. oryzae. Additionally, the conidia undergo one round of mitosis followed by autophagic nuclear degradation during appressoria formation in M. oryzae (45, 66, 70). Here, of particular interest, we found that the conidia of *C. fructicola* is accompanied by multiple rounds of mitosis and no nuclear degradation during appressoria formation, again indicating a specific difference between distinct appressoria-forming phytopathogens. How mitosis functions in infection-related development in *C. fructicola* remains unknown, and further studies are warranted.

In summary, we showed that the nucleus-localized CfGcn5 regulates global gene expression, including that of multiple *ATG* genes. We confirmed that CfGcn5 is a negative regulator of autophagy and undergoes degradation when pathogenicity-related autophagy occurs. Further studies will focus on the targets of CfGcn5, both in transcription and in acetylation, to elucidate the regulatory network of CfGcn5-mediated autophagy governing pathogenicity in *C. fructicola*.

## MATERIALS AND METHODS

### Strains and culture conditions.

The CFLH16 strain was used as the WT of *C. fructicola*. All strains were cultured on either PDA or MM medium in darkness at 28°C as previously described (7). Liquid PDB was applied to the culture strains for conidia as well as DNA, RNA, and protein extractions.

### Transcriptional sequencing and analysis.

Total RNA was extracted from the WT and Δ*Cfgcn5* mutant cultured on liquid PDB for 2 days. Each strain contains three biological replicates. The total RNA was quality controlled, purified, and reverse transcribed to cDNA. Then, the cDNA library was further cleaned and sequenced using the Illumina Hi-Seq platform, and 150 bp paired-end reads were generated. The paired-end clean reads were aligned to the reference genome of *C. fructicola* using Hisat2 (71). DEGs were analyzed with by DESeq2 (72). Genes with an adjusted *P* < 0.01 and a fold change of ≥2 found by DESeq2 were assigned as DEGs, which were then further analyzed by GO and KEGG enrichment analyses.

### Growth and stress response assays.

For the growth assays, small mycelial plugs were cut from the edge of a 3-day-old colony and cultured on either PDA or MM agar plates for 4 days, followed by a measurement and a statistical analysis of the colony diameter. For the stress response assays, the concentration of 25 nM rapamycin was added to PDA as a stress inducer, and the inhibition rates of the colony diameter were statistically analyzed.

### qRT-PCR and gene expression assays.

Total RNA was extracted and reverse-transcribed into first-strand cDNA using the Reverse Transcription kit (Vazyme). The qRT-PCR with primers (Table S2) was run on an ABI QuantStudio 3, and the relative quantification of transcription was normalized to the stable expression *ACTIN* gene. The experiments were repeated three times, using three independent biological replicates each time.

10.1128/mbio.01956-22.7 TABLE S2 Primers used in this study. Download Table S2, DOC file, 0.02 MB. Copyright © 2022 Zhang et al. 2022 Zhang et al. https://creativecommons.org/licenses/by/4.0/ This content is distributed under the terms of the Creative Commons Attribution 4.0 International license.

### Generation of the CfGcn5^E129Q^-GFP, CfGcn5^T167AY168A^-GFP, CfAtg8-GFP, CfAtg9-GFP, P*_RP27_*-GFP-CfAtg8, P*_RP27_*-GFP-CfAtg9, CfApe1-RFP, and H1-RFP constructs.

For generating CfGcn5^E129Q^-GFP, CfGcn5^T167AY168A^-GFP, CfAtg8-GFP, and CfAtg9-GFP, the ~1.5 kb native promoter and the full-length or amino acid substituted fragments were inserted into a pYF11 vector as previously described (73). For generating P*_RP27_*-GFP-CfAtg8 and P*_RP27_*-GFP-CfAtg9, the GFP and the full-length of CfAtg8 or CfAtg9 were first fused together and then inserted into the pYF11 vector. For generating CfApe1-RFP and H1-RFP, the ~1.5 kb native promoter and the full-length of CfApe1 or H1 were inserted into a pHZ126 vector.

### Pathogenicity assays.

The droplets (20 μL) of conidial suspensions (3 × 10^5^ spores/mL) or mycelial plugs (3 mm × 3 mm) were inoculated on the edges of healthy or wounded *C. oleifera* leaves and incubated on high-humidity plates for 4 days. The lesions were observed and analyzed with ImageJ. For the pathogenicity on the apples, we first punched several 8-mm-diameter holes in their epidermises, and mycelial plugs (3 mm × 3 mm) were then inoculated into the holes. After incubation for 5 to 7 days, the lesions (total area minus hole area) were analyzed by ImageJ.

### Localization observation.

The hypha, conidia, germ tubes, and appressoria expressing GFP or RFP fused proteins were observed under fluorescence microscopy (ZEISS, Axio Observer. A1). CfApe1-RFP and H1-RFP were introduced to mark the autophagosomes and the nuclei, respectively.

### Protein extraction and Western blot analysis.

The mycelia of strains were cultured in liquid PDA under 180 rpm at 28°C for 36 h, washed with ddH_2_O, and moved to MM-N medium under 80 rpm at 28°C for 2 h and 5 h. The mycelia were further collected and ground into powder in liquid nitrogen and then resuspended in 1 mL RIPA lysis buffer (EpiZyme, PC101) with 10 μL of a proteinase inhibitor cocktail (EpiZyme, GRF101). The lysates were incubated in ice for 30 min, resuspended with a Vortex-Genie every 10 min, and further centrifuged at 12,000 × *g* for 20 min at 4°C. The supernatant proteins were harvested. The proteins were analyzed by 10% SDS-PAGE followed by Western blotting with the primary antibodies being anti-GFP (rabbit, 1:5,000, Abways, AB0045), anti-α-H3K18ac (rabbit, 1:3,000, Beyotime, AF5617), anti-α-H3 (rabbit, 1:2,000, Cell Signaling Technology, 4499), and anti-β-tubulin (mouse, 10,000, Engibody, AT0003) and the secondary antibodies being HRP-labeled goat anti-rabbit lgG (H+L) (1:10,000, Abways, AB0101) and HRP-labeled goat anti-mouse lgG (H+L) (1:20,000, HUABIO, HA1006), respectively. Finally, the proteins were detected by an Omni-ECL Femto Light Chemiluminescence Kit (EpiZyme, SQ201) and analyzed by ImageJ.

### Gene deletion, complementation, and amino acid substitution.

Targeted gene deletion was used as the one-step replacement strategy, as in our previous description (63). First, two ~1.0 kb sequences flanking the targeted gene were amplified and overlapped with the flanks of *HPH* (hygromycin resistance cassette). Then, the resulting ~3.4 kb fragments were introduced into protoplasts of the WT. For complementation and amino acid substitution, the constructed vectors were introduced into protoplasts of specific mutant strains.

### Appressoria formation and turgor pressure assays.

The conidia were harvested and filtered by lens paper and then washed with ddH_2_O twice. After being resuspended to a concentration of 3 × 10^5^ spores/mL, the conidial suspensions (20 μL) were dripped onto hydrophobic coverslips (Fisher Scientific) and observed at a series of time intervals. The turgor pressure was measured via a cytorrhysis assay (50). The water in the 24 h appressoria was removed and substituted by a 4 M glycerol solution (20 μL). After 10 min of incubation, the appressoria of cytorrhysis and plasmolysis were observed and statistically analyzed.

### Statistical analysis.

All data were presented as mean ± standard deviation and analyzed using a one-way analysis of variance (ANOVA) followed by Duncan’s new multiple range test, *P* < 0.01 or *P* < 0.05.

### Accession numbers.

The GenBank accession numbers of the sequence data in this article are as follows: MW701426 (CfGcn5), MK622895 (CfAtg8), MK622896 (CfAtg9), NP_011768.1 (ScGcn5), XP_003716207.1 (MoGcn5), EQB51886.1 (CgGcn5), XP_006966860.1 (TrGcn5), XP_023425542.1 (FfGcn5), XP_011324943.1 (FgGcn5), XP_001728480.2 (NcGcn5), and XP_661225.1 (AnGcn5). All of the raw data from the RNA-seq are available from the SRA database with accession numbers SRR19052615, SRR19052614, and SRR19052613 for WT 1 to 3 and SRR19052621, SRR19052620, and SRR19052619 for Δ*Cfgcn5* 1 to 3.
