# Supplementary material for: Histone Acetyltransferase CfGcn5-Mediated Autophagy Governs the Pathogenicity of Colletotrichum fructicola
Source: mBio. 2022 Aug 17;13(5):e01956-22. doi: 10.1128/mbio.01956-22 (PMC9600425; doi:10.1128/mbio.01956-22)
Supplement: TABLE S1 [file mbio.01956-22-s0005.doc]

**Table S1. The expression levels of *CfATG* genes in transcriptome data**

| Gene ID | Gene name | Δ*Cfgcn5*/WT | P value |
| --- | --- | --- | --- |
| gene-CGGC5_v001583 | *CfATG1* | 0.82 | 0.09 |
| gene-CGGC5_v006476 | *CfATG2* | 0.61 | 1.75E-06 |
| gene-CGGC5_v011295 | *CfATG3* | 1.53 | 7.49E-05 |
| gene-CGGC5_v013845 | *CfATG4* | 2.20 | 3.83E-14 |
| gene-CGGC5_v006481 | *CfATG5* | 0.93 | 0.66 |
| gene-CGGC5_v012401 | *CfATG6* | 1.29 | 0.006 |
| gene-CGGC5_v015799 | *CfATG7* | 1.03 | 0.85 |
| gene-CGGC5_v010022 | *CfATG8* | 0.81 | 0.10 |
| gene-CGGC5_v013854 | *CfATG9* | 1.34 | 0.01 |
| gene-CGGC5_v001121 | *CfATG10* | 1.52 | 0.004 |
| gene-CGGC5_v014758 | *CfATG12* | 1.80 | 6.35E-07 |
| gene-CGGC5_v014851 | *CfATG13* | 1.06 | 0.59 |
| gene-CGGC5_v007470 | *CfATG15* | 0.68 | 0.004 |
| gene-CGGC5_v001805 | *CfATG16* | 1.18 | 0.16 |
| gene-CGGC5_v008931 | *CfATG18* | 1.49 | 0.007 |
