# Supplementary figures and images for: Histone Acetyltransferase CfGcn5-Mediated Autophagy Governs the Pathogenicity of Colletotrichum fructicola
Source: mBio. 2022 Aug 17;13(5):e01956-22. doi: 10.1128/mbio.01956-22 (PMC9600425; doi:10.1128/mbio.01956-22)

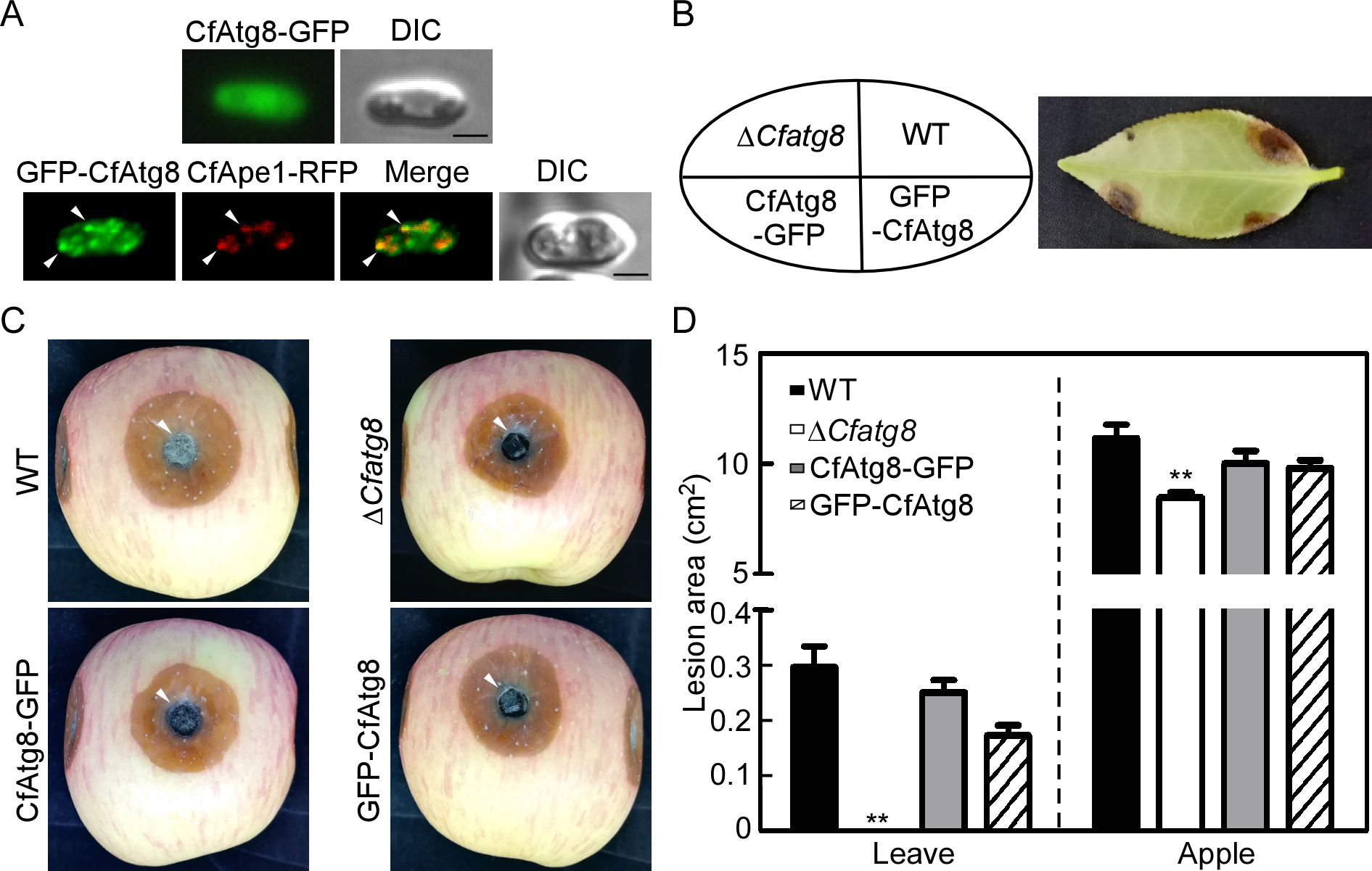

Supplement: FIG S1 [file mbio.01956-22-s0001.tif]

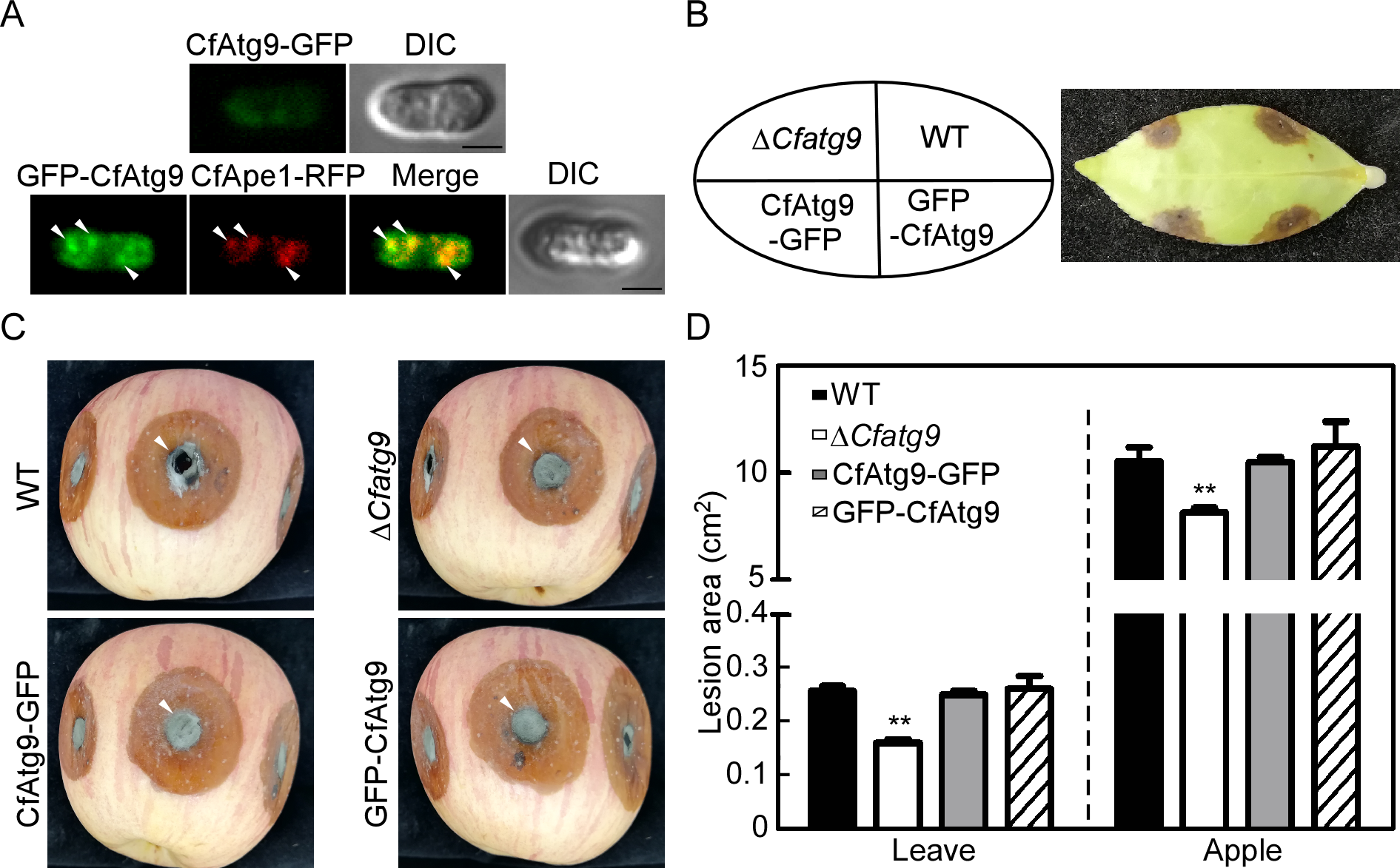

Supplement: FIG S4 [file mbio.01956-22-s0004.tif]
