# Supplementary material for: Histone Acetyltransferase CfGcn5-Mediated Autophagy Governs the Pathogenicity of Colletotrichum fructicola
Source: mBio. 2022 Aug 17;13(5):e01956-22. doi: 10.1128/mbio.01956-22 (PMC9600425; doi:10.1128/mbio.01956-22)
Supplement: TABLE S2 [file mbio.01956-22-s0006.doc]

**Table S2 Primers used in this study**

| **Primer name** | **Sequence (5’-3’)** | **Remark** |
| --- | --- | --- |
| qRT1F | ATGAACGGCACCCATAACG | qRT-PCR Primer of v009786 |
| qRT1R | CCAGGTCCAGACGCTGAGAT | qRT-PCR Primer of v009786 |
| qRT2F | CGAGACCATTGCGACCAAC | qRT-PCR Primer of v013155 |
| qRT2R | GCCCTGAACAGCGAAGAAG | qRT-PCR Primer of v013155 |
| qRT3F | TCGCCATTGTTCAGGGAGG | qRT-PCR Primer of v002571 |
| qRT3R | AGTTGGTGGACGGGGGTAT | qRT-PCR Primer of v002571 |
| qRT4F | CGCACACCCGATACCTACA | qRT-PCR Primer of v005151 |
| qRT4R | AGCCAGCATTACCGAAACC | qRT-PCR Primer of v005151 |
| qRT5F | TCCAGATTGCCTCCATCCTCG | qRT-PCR Primer of v011727 |
| qRT5R | CCGACATCAGGCTTGACACAGG | qRT-PCR Primer of v011727 |
| qRT6F | CCGATATGGGCGGAATTATGT | qRT-PCR Primer of v014591 |
| qRT6R | CTTCTTGGCGGCGTTGTGG | qRT-PCR Primer of v014591 |
| qRT7F | ATCATCACCCCTCCCCCCACCA | qRT-PCR Primer of v002744 |
| qRT7R | TTGCCACCCATGCCGCCAGTAG | qRT-PCR Primer of v002744 |
| qRT8F | ACTCCGACGCTTCCCTCACC | qRT-PCR Primer of v003631 |
| qRT8R | GGCGGACTCCTTGTGGACAT | qRT-PCR Primer of v003631 |
| qRT9F | TCAGTCGGAGATGTCAGGTTT | qRT-PCR Primer of v005562 |
| qRT9R | GCTCAGGGATAATGCGGTAG | qRT-PCR Primer of v005562 |
| qRT10F | CGACATCTGGCGTTGCTAAT | qRT-PCR Primer of v005654 |
| qRT10R | TGAGAGGCGGAGGAGACTTG | qRT-PCR Primer of v005654 |
| qRT*CfATG1*F | AAACTGGGGACGGACTTGG | qRT-PCR Primer of *CfATG1* |
| qRT*CfATG1*R | ATCCTGAGCCGCCTTTTCT | qRT-PCR Primer of *CfATG1* |
| qRT*CfATG2*F | CAAGAACTCCCTCGAAACAACA | qRT-PCR Primer of *CfATG2* |
| qRT*CfATG2*R | GCTCAATCCGCCAAACCAG | qRT-PCR Primer of *CfATG2* |
| qRT*CfATG3*F | GAGGATTTCCCCTTCTTCGC | qRT-PCR Primer of *CfATG3* |
| qRT*CfATG3*R | CTGGTCGTCAACCTCGCTTT | qRT-PCR Primer of *CfATG3* |
| qRT*CfATG4*F | TGGGTCGGTCATACAATCT | qRT-PCR Primer of *CfATG4* |
| qRT*CfATG4*R | TCCATCTTGAACAGCGTAATC | qRT-PCR Primer of *CfATG4* |
| qRT*CfATG5*F | CCCGACGCTGTTCCCATCCA | qRT-PCR Primer of *CfATG5* |
| qRT*CfATG5*R | CGACCGTGACACAGAGCCAACC | qRT-PCR Primer of *CfATG5* |
| qRT*CfATG6*F | CACTCCTCACGAATCCCACA | qRT-PCR Primer of *CfATG6* |
| qRT*CfATG6*R | CCTTTTGAACCAGCCTTGC | qRT-PCR Primer of *CfATG6* |
| qRT*CfATG7*F | TCGGCTTGGTTCCTCACAC | qRT-PCR Primer of *CfATG7* |
| qRT*CfATG7*R | CAGCACCCTCCTCTTCATCTT | qRT-PCR Primer of *CfATG7* |
| qRT*CfATG8*F | CCGATCTCCCATCATCCCG | qRT-PCR Primer of *CfATG8* |
| qRT*CfATG8*R | TGCTCCTCGTAGATGCTGCTC | qRT-PCR Primer of *CfATG8* |
| qRT*CfATG9*F | ATTTTGGTCTTTGGCTGTATGT | qRT-PCR Primer of *CfATG9* |
| qRT*CfATG9*R | CTCTTTGGATTGGTTCCGTAT | qRT-PCR Primer of *CfATG9* |
| qRT*CfATG10*F | TGCCACCACTTTGACAGCC | qRT-PCR Primer of *CfATG10* |
| qRT*CfATG10*R | TTCAGCATCTATCATAGCCTCGT | qRT-PCR Primer of *CfATG10* |
| qRT*CfATG12*F | TAAGTTCAAGCCCGTGGG | qRT-PCR Primer of *CfATG12* |
| qRT*CfATG12*R | GCCAAAGGCTGGGTTCATAG | qRT-PCR Primer of *CfATG12* |
| qRT*CfATG13*F | AAAACGATACCCACCAACG | qRT-PCR Primer of *CfATG13* |
| qRT*CfATG13*R | ATACAGCGGATGCCGAAGC | qRT-PCR Primer of *CfATG13* |
| qRT*CfATG15*F | GGGATGTTTGGACGAGACG | qRT-PCR Primer of *CfATG15* |
| qRT*CfATG15*R | GAGGTGATGAGATGCGAAGTG | qRT-PCR Primer of *CfATG15* |
| qRT*CfATG16*F | AACTCTCCGACCGCATCGC | qRT-PCR Primer of *CfATG16* |
| qRT*CfATG16*R | ATCTGCTTCGTGTTCTCCCTGT | qRT-PCR Primer of *CfATG16* |
| qRT*CfATG18*F | ACAGTGACGGAAATGTGGGA | qRT-PCR Primer of *CfATG18* |
| qRT*CfATG18*R | AACCGAGAGCTGCTTGACC | qRT-PCR Primer of *CfATG18* |

| qRT*CfGCN5F* | AAGGATTACGAAGGAGGCACA | qRT-PCR Primer of Cf*GCN5* |
| --- | --- | --- |
| qRT*CfGCN5R* | AGGTCGTTGAGGAGGTGGAG | qRT-PCR Primer of Cf*GCN5* |

| qRT*ACTIN*F | ATCAACCCCAAGTCCAACAG | qRT-PCR Primer of *ACTIN* |
| --- | --- | --- |
| qRT*ACTIN*R | CGATTTCACGCTCGGCAGT | qRT-PCR Primer of *ACTIN* |
| Gcn5GFPF | ACTCACTATAGGGCGAATTGGGTACTCAAATTGGTTCTGGACGGTAATTGCGGATGT | Construction of CfGcn5^E129Q^-GFP |
| E129QR1 | GGCGAACTGGCGGCCTTTG | Construction of CfGcn5^E129Q^-GFP |
| E129QF2 | CAAAGGCCGCCAGTTCGCCCAAATCGTTTTCTGTGCTAT | Construction of CfGcn5^E129Q^-GFP |
| Gcn5GFPR | CACCACCCCGGTGAACAGCTCCTCGCCCTTGCTCACCGGTTCGAGGTGAGACCACTC | Construction of CfGcn5^E129Q^-GFP |
| T167AY168AR1 | GAGGAAGTGCATGACGTCG | Construction of CfGcn5^T167AY168A^-GFP |
| T167AY168AF2 | CGACGTCATGCACTTCCTCGCCGCCGCCGACAACTATGCCAT | Construction of CfGcn5^T167AY168A^-GFP |
| CfAtg8GFPF | ACTCACTATAGGGCGAATTGGGTACTCAAATTGGTTCTGCCAGCCCCAGAGCGACCCTT | Construction of CfAtg8-GFP |
| CfAtg8GFPR | CACCACCCCGGTGAACAGCTCCTCGCCCTTGCTCACCGCCGTCTCGAAACCGCCGAAG | Construction of CfAtg8-GFP |
| CfAtg8NGFPF2 | GCATGGACGAGCTGTACAAGATGCGATCCAAGTTCAAGG | Construction of P*_RP27_*-GFP-CfAtg8 |
| CfAtg8NGFPR2 | CACCACCCCGGTGAACAGCTCCTCGCCCTTGCTCACTCACGCCGTCTCGAAACCGCCG | Construction of P*_RP27_*-GFP-CfAtg8 |
| CfAtg8rp27NGFPF | TTTCGTAGGAACCCAATCTTCAAAATGGTGAGCAAGGGCGAGG | Construction of P*_RP27_*-GFP-CfAtg8 |
| NGFPR | CTTGTACAGCTCGTCCATGC | Construction of P*_RP27_*-GFP-CfAtg8 |
| CfAtg9GFPF | ACTCACTATAGGGCGAATTGGGTACTCAAATTGGTTACCGTTACTGTCAATAACCTC | Construction of CfAtg9-GFP |
| CfAtg9GFPR | CACCACCCCGGTGAACAGCTCCTCGCCCTTGCTCACAAGTGCTCCACTTTGGCGATC | Construction of CfAtg9-GFP |
| CfAtg9NGFPF2 | GCATGGACGAGCTGTACAAGATGGCTTCCAACATCTTCTC | Construction of P*_RP27_*-GFP-CfAtg9 |
| CfAtg9NGFPR2 | CACCACCCCGGTGAACAGCTCCTCGCCCTTGCTCACTTAAAGTGCTCCACTTTGGCG | Construction of P*_RP27_*-GFP-CfAtg9 |
| CfAtg9UF | AATCCGCCGTTGACCAGGAG | amplify *CfATG9* 5’ flank sequence |
| CfAtg9UR | TTGACCTCCACTAGCTCCAGCCAAGCCGCTGACTGTGTGGTGCAACAAGAAT | amplify *CfATG9* 5’ flank sequence |
| CfAtg9DF | CAAAGGAATAGAGTAGATGCCGACCGGGTGCAGTGAGGATAGACTACTGCC | amplify *CfATG9* 3’ flank sequence |
| CfAtg9DR | ACAGACGACCCGTCATCGA | amplify *CfATG9* 3’ flank sequence |
| CfAtg9UFG418 | AATCCGCCGTTGACCAGGAG | amplify *CfATG9* 5’ flank sequence for Δ*Cfgcn5*Δ*Cfatg9* |
| CfAtg9URG418 | CAATATCATCTTCTGTCGACGCTGACTGTGTGGTGCAACAAGAAT | amplify *CfATG9* 5’ flank sequence for Δ*Cfgcn5*Δ*Cfatg9* |
| CfAtg9DFG418 | TTCTTGACGAGTTCTTCTGAGGTGCAGTGAGGATAGACTACTGCC | amplify *CfATG9* 3’ flank sequence for Δ*Cfgcn5*Δ*Cfatg9* |
| CfAtg9DRG418 | ACAGACGACCCGTCATCGA | amplify *CfATG9* 3’ flank sequence for Δ*Cfgcn5*Δ*Cfatg9* |
| CfAtg9BWF | ATGTAGGCGTTGGGGGAGGAG | validation of *CfATG9* deletion |
| HPHR | CGCTACTGCTACAAGTGGGGCT | validation of *CfATG9* deletion |
| G418R | TCAGAAGAACTCGTCAAGAA | validation of *CfATG9* deletion |
| H1RFPF | CTATAGGGCGAATTGGGTACTCAAATTGGTTGTCCAGGGTGTGACAGCAGA | Construction of H1-RFP |
| H1RFPR | GAACTCCTTGATGACGTCCTCGGAGGAGGCCGCAGATGCGGCAGCGGGCT | Construction of H1-RFP |
| CfApe1RFPF | CTATAGGGCGAATTGGGTACTCAAATTGGTTCTCGCCAACTACGCACCCA | Construction of CfApe1-RFP |
| CfApe1RFPR | GAACTCCTTGATGACGTCCTCGGAGGAGGCGTGCCACTCGCCGTCAATCTT | Construction of CfApe1-RFP |
